# Supplementary material for: The KMT2F histone methyltransferase interacts with the RNA polymerase I machinery to promote ribosomal RNA transcription
Source: PLoS Biol. 2026 May 7;24(5):e3003785. doi: 10.1371/journal.pbio.3003785 (PMC13178980; doi:10.1371/journal.pbio.3003785)
Supplement: S1 Fig — (PDF) [file pbio.3003785.s001.pdf]

Supplementary Figure 1: Endogenous KMT2A and KMT2F localize to the nucleolus.

A,B. Immunoblotting and immunofluorescence staining (IFS) analyses show loss of nucleolar localization of KMT2A and KMT2F following RNAi-mediated depletion. U-2OS cells were transfected with control, KMT2A-specific, or KMT2F-specific siRNAs, as indicated. Protein depletion was confirmed by immunoblotting. Cells were fixed and co-immunostained with antibodies against KMT2A or KMT2F and the nucleolar marker B23. Nuclear DNA was counterstained with 4′,6-diamidino-2-phenylindole (DAPI; blue). Scale bar, 5 µm. The uncropped blots shown in A-B can be found in S1 Raw Images.

C,D. Immunoblot and IFS reveal loss of nucleolar localization of KMT2A and KMT2F upon RNAi-mediated depletion. HEK293 cells were treated with control, KMT2A, or KMT2F siRNAs and co-stained with KMT2A/fibrillarin or KMT2F/UBF antibodies. DNA was stained with DAPI (blue). Scale bar, 5 µm. The uncropped blots shown in C-D can be found in S1 Raw Images.

E,F. Nucleolar localization of endogenous KMT2A (E) and KMT2F (F) is shown in three different cell lines viz: IMR90-tert, HeLa and MCF-7. Cells were co-stained with KMT2A or KMT2F and B23 (DAPI, blue). Scale bar, 5 µm

G. Images of GFP-KMT2A and GFP-KMT2F co-expressed with dsRed-B23 in HEK293 cells are shown. Nuclei were counterstained with DAPI. White arrows highlight the nucleolus.

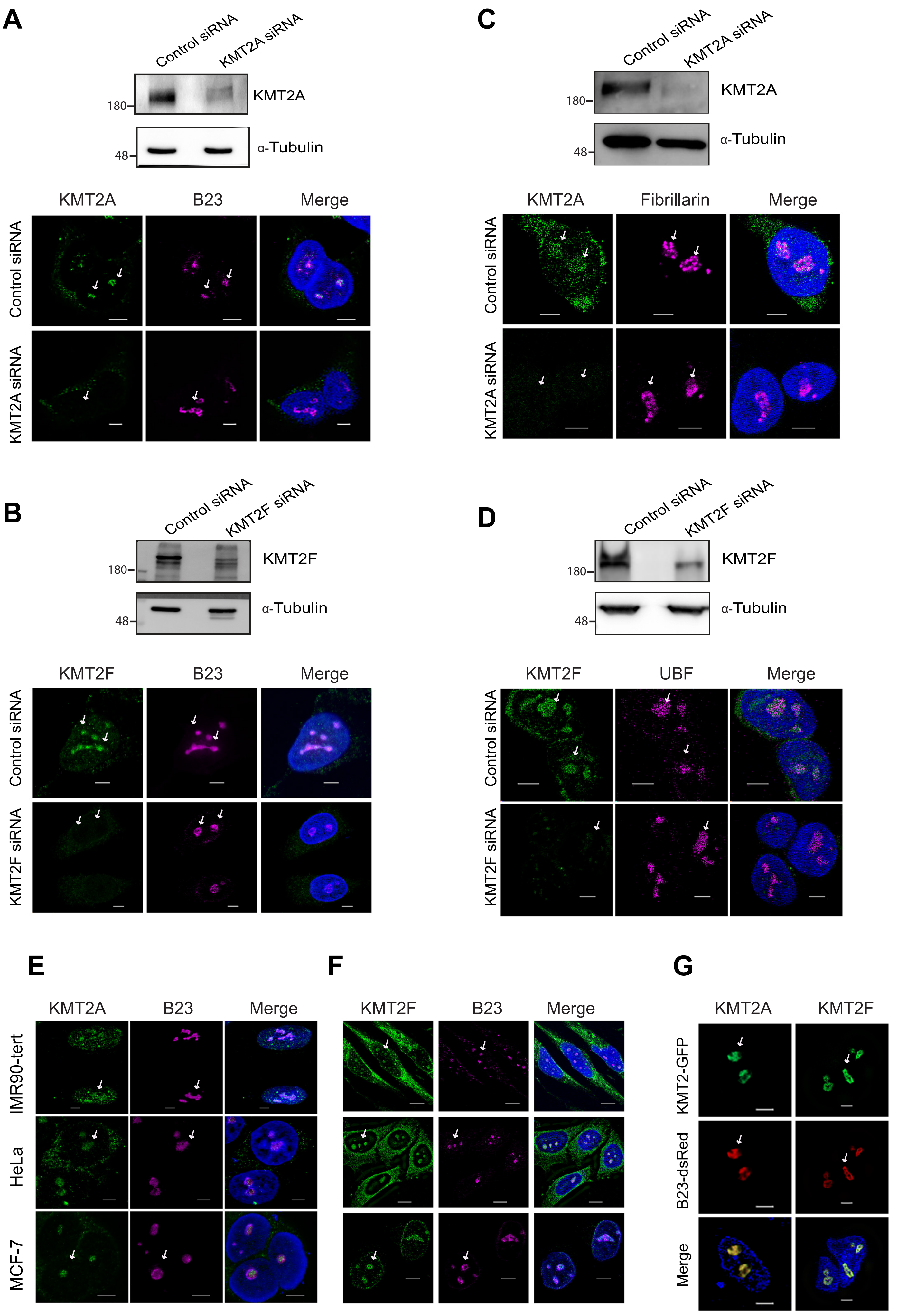

Lone Supplementary Figure 1
