# Supplementary material for: The KMT2F histone methyltransferase interacts with the RNA polymerase I machinery to promote ribosomal RNA transcription
Source: PLoS Biol. 2026 May 7;24(5):e3003785. doi: 10.1371/journal.pbio.3003785 (PMC13178980; doi:10.1371/journal.pbio.3003785)
Supplement: S2 Fig — (PDF) [file pbio.3003785.s002.pdf]

Supplementary Figure 2: ChIP-seq analysis of human rDNA.

Chromatin immunoprecipitation-sequencing (ChIP-seq) maps illustrating the binding of RNA Pol I (RPA116), UBF, WDR82, WDR5, KMT2A, KMT2F, H3K4me2 and H3K4me3 at the entire human rDNA locus are shown along with DOT1Land H3K79me2. ChIP enrichment for each factor is expressed as the ratio of immunoprecipitated ChIP-seq signal to Input DNA ChIP-seq signal. The vertical scale in each panel represents the enrichment of each component relative to the input DNA dataset. In the assembly annotation, Subin et al. (Subin et al., 2023) excised 9.18 kb from the end of the rDNA repeat and attached it to the beginning of the repeat. As a result, the 47S promoter lies at 9.18 kb from start. Therefore, the scale shows — 9.18 to 34.8 kb with 47S promoter denoted as 0. Inset of dashed lines is shown in Figures 1B and 2A. The underlying ChIP-seq data can be found under Accession numbers SRP004987 (Pol I & UBF), GSE198645 (H3K4me2/me3), GSE261933 (KMT2A/F), GSE186758 (WDR82), GSE60897 (WDR5), GSE149484 (H3K79me2), DRA004872 (DOT1L) .

ChIP-seq signals

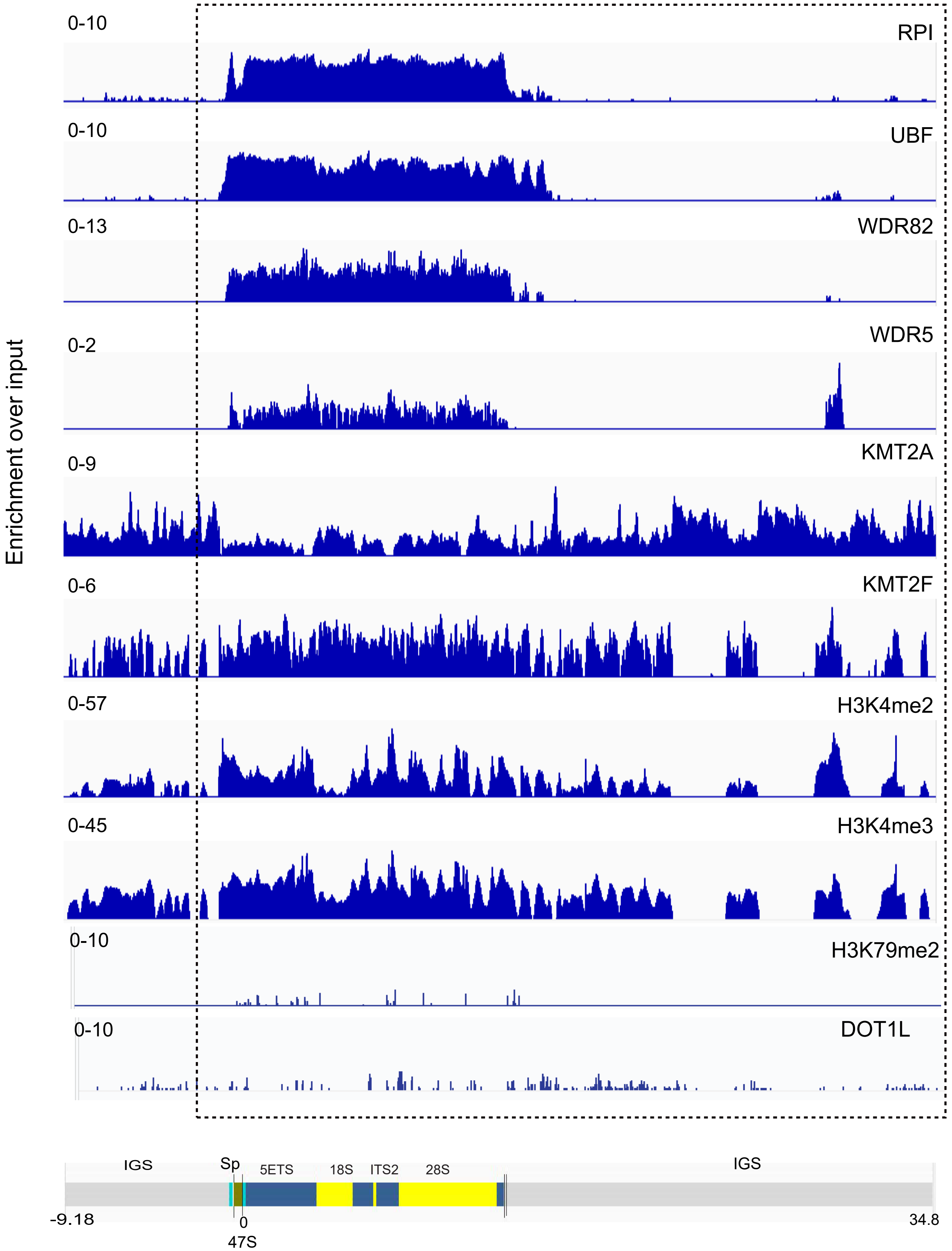

Lone Supplementary Figure 2
