# Supplementary material for: The KMT2F histone methyltransferase interacts with the RNA polymerase I machinery to promote ribosomal RNA transcription
Source: PLoS Biol. 2026 May 7;24(5):e3003785. doi: 10.1371/journal.pbio.3003785 (PMC13178980; doi:10.1371/journal.pbio.3003785)
Supplement: S3 Fig — (PDF) [file pbio.3003785.s003.pdf]

Supplementary Figure 3. KMT2 and RNA Pol I bind to the human rDNA repeats.

A-E. ChIP of RNA Pol I (A), UBF (B), KMT2A (C), KMT2F (D), H3(E) followed by qRT-PCR analysis is shown. KMT2A and KMT2F ChIP were performed in IMR-90 tert cells, whereas RNA Pol I, UBF, and H3 ChIP were performed in HEK-293 cells. Primers 1-18 were used to assess the binding of KMT2A/KMT2F over the 43kb human rDNA repeat. Spacer promoter, terminator element (T0) and 47S (core) promoter are represented by primers 1- 3; transcribed region: 4-8 and IGS by 9-18. HOXA9 and RAD18 were used as positive control regions for KMT2A and KMT2F binding, respectively, while CD4 was used as a negative control. RNA Pol I and UBF binding was analyzed in the promoter and in the coding region of rDNA (primers 1 to 8, primer 9 as control). Data is represented from three or more than three experiments. Error bars represent SD. Significance in A & B was calculated for each primer pair with respect to primer #9, while in C-E, significance was calculated with respect to the CD4 using One-way ANOVA with Dunnett's multiple comparison test. $*P \leq 0.05$, $** P \leq 0.005$, $***P \leq 0.0005$, $****P \leq 0.00005$. Green: RNA Pol I promoter; blue: RNA Pol I transcribed region; orange: IGS. The underlying raw data pertaining to A-E can be found in S1 Data.

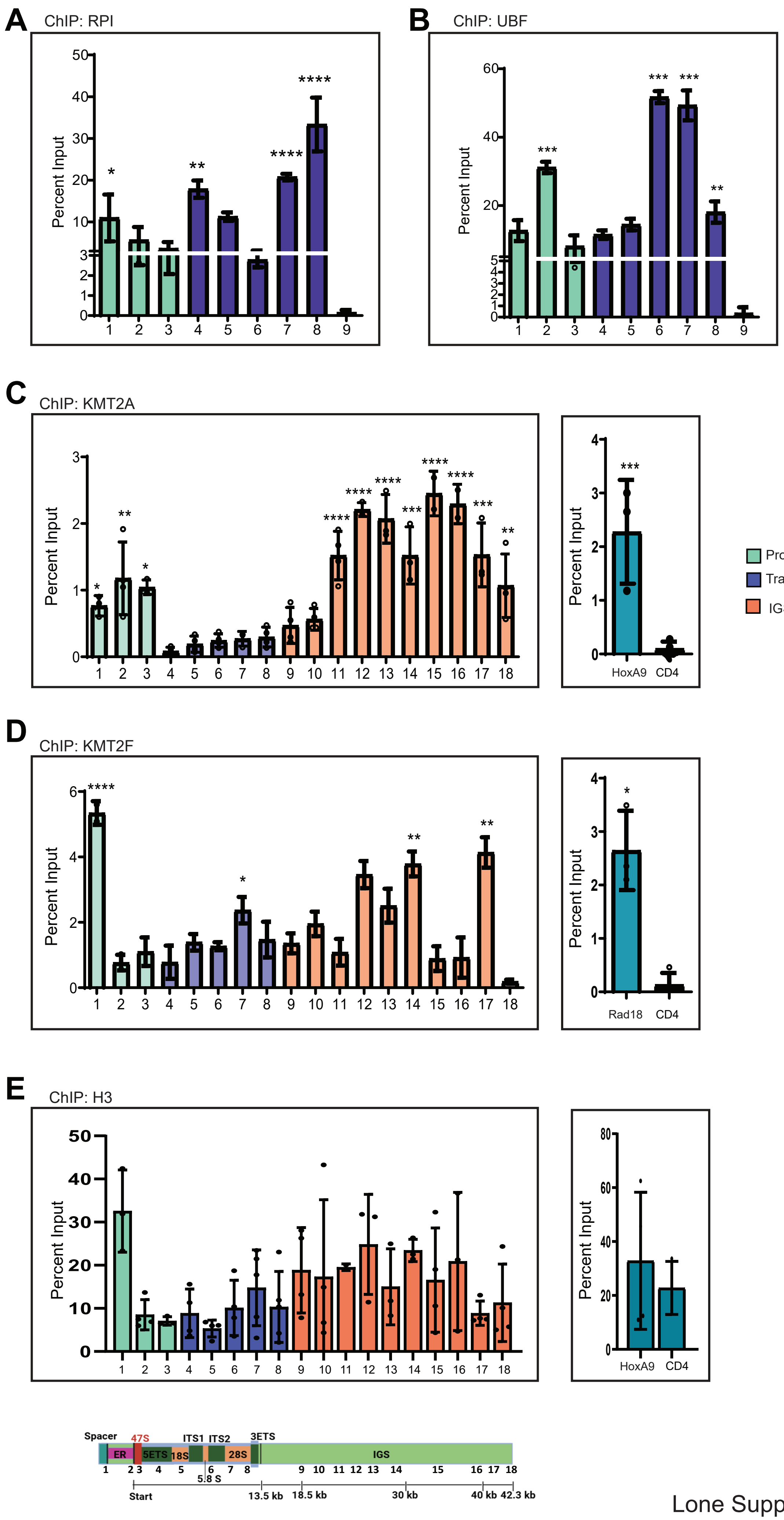

Lone Supplementary Figure 3
