# Supplementary material for: The KMT2F histone methyltransferase interacts with the RNA polymerase I machinery to promote ribosomal RNA transcription
Source: PLoS Biol. 2026 May 7;24(5):e3003785. doi: 10.1371/journal.pbio.3003785 (PMC13178980; doi:10.1371/journal.pbio.3003785)
Supplement: S4 Fig — (PDF) [file pbio.3003785.s004.pdf]

Supplementary Figure 4: Correlation between ChIP-seq and ChIP-qPCR data.

A-D. Primer-specific read counts derived from ChIP-seq datasets for KMT2A (A), KMT2F (B), H3K4me2 (C), and H3K4me3 (D) were quantified at rDNA regions corresponding to the ChIP-qPCR amplicons and directly compared with ChIP-qPCR enrichment values (shown in Fig.1D-E and 2B-C) obtained using the same primer sets. The ChIP-seq signal obtained for each primer set was normalized to its respective amplicon size and then expressed as reads per kilobase per million reads mapped (RPKM). Reads plotted as RPKM for each primer are aligned with the respective ChIP-qPCR of each primer in the form of a line graph. The underlying raw data pertaining to A-D can be found in S1 Data.

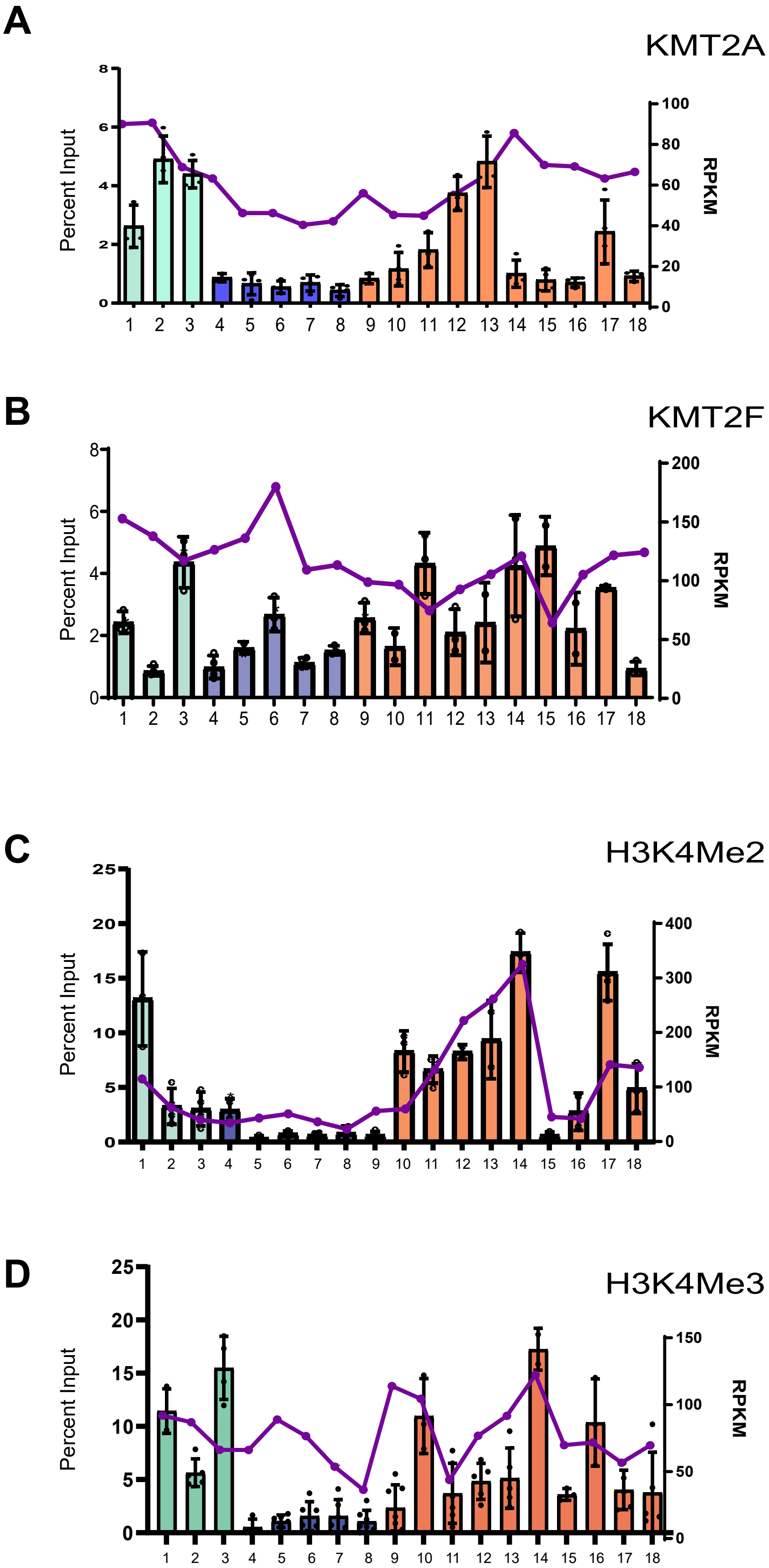

Lone Supplementary Figure 4
