# Supplementary material for: The KMT2F histone methyltransferase interacts with the RNA polymerase I machinery to promote ribosomal RNA transcription
Source: PLoS Biol. 2026 May 7;24(5):e3003785. doi: 10.1371/journal.pbio.3003785 (PMC13178980; doi:10.1371/journal.pbio.3003785)
Supplement: S5 Fig — The second part describes the effect of KMT2A and KMT2F depletion on ribosome biogenesis as assessed by RNA-seq. (PDF) [file pbio.3003785.s005.pdf]

Supplementary Figure 5: Profiling of KMT2A and KMT2F-regulated genes reveals a coordinated epigenetic and transcriptional regulation of rDNA.

A-F. Genome-wide ChIP-seq analysis showing binding of RNA polymerase I (A), UBF (B), KMT2A (C), KMT2F (D), H3K4me2 (E), and H3K4me3 (F) across the genome, including enrichment at rDNA loci. Distribution of each factor on the various regions of the genome, like on the Promoters, Gene body, Intergenic regions and on the rDNA, was quantified using Multi Bam coverage as discussed in the material and methods. The distribution in these different regions of the genome for each factor was plotted in the form of Venn diagram as a percentage.

G-H. GO enrichment analysis of differentially regulated transcripts identified in KMT2A (G) and KMT2F (H) siRNA-treated cells. Biological process GO terms were ranked based on adjusted *P*-values obtained using the GSEA R package. The most significantly enriched positively and negatively regulated GO terms are shown. In addition, terms associated with ribosome biogenesis, which are of interest here, are shown even though they are neither significant nor enriched in these datasets (see zero NES). Colour indicates the normalized enrichment score (NES) as shown.

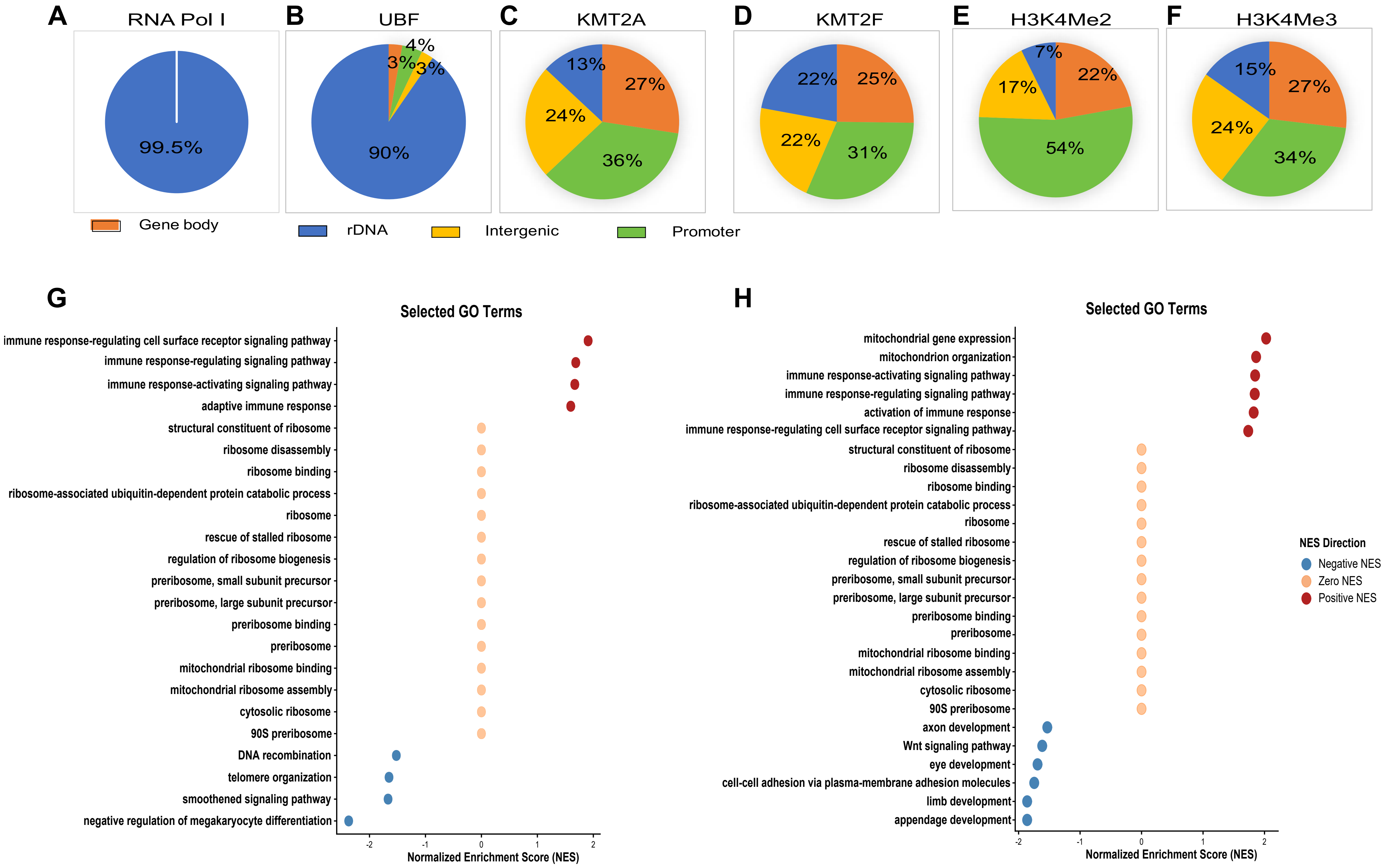

Lone Supplementary Figure 5
