# Supplementary material for: The KMT2F histone methyltransferase interacts with the RNA polymerase I machinery to promote ribosomal RNA transcription
Source: PLoS Biol. 2026 May 7;24(5):e3003785. doi: 10.1371/journal.pbio.3003785 (PMC13178980; doi:10.1371/journal.pbio.3003785)
Supplement: S6 Fig — (PDF) [file pbio.3003785.s006.pdf]

Supplementary Figure 6: RNAi-mediated depletion of KMTs affects ribosomal DNA transcription.

A. HeLa cells were subjected to endogenous immunoprecipitation (IP) using an antibody against the N-terminal subunit of KMT2A. The anti-immunoglobulin G (IgG)-mock antibody was used as a control. The immunoblot was probed with α-RPA194, α-UBF, α-RRN3, and α-KMT2A antibodies.

B. Cells were subjected to endogenous IP using antibodies against KMT2A or KMT2F, with anti-IgG used as a mock control. Immunoblots were probed with antibodies against RNA polymerase II, RPA194 (RNA polymerase I), KMT2A, and KMT2F. A-B, Molecular weight markers are indicated on the left.

C. Represents the immunoblots of WDR5 (a) KMT2A (b) KMT2B (c) and KMT2F (d) in the control and siRNA-treated U2OS cells. Immunoblots were probed with the respective antibodies, and Alpha-tubulin used as a loading control.

D-E. Represents the q-PCR analysis of KMT2A and KMT2F (D) transcripts in siRNA-depleted HEK293 cells while (E) represents the levels of 5ETS in the same cells. Significance was calculated with respect to the control sample using Students t-test. Error bars denote SD. *P ≤ 0.05, ** P ≤ 0.005, ***P≤ 0.0005, ns: not significant P > 0.05 (two-tailed Students t test).

F. Schematic of KMT2A and KMT2A mutant used in transcriptional analysis in Figure 4 are shown. Recombinant full-length KMT2A (FL) was generated by PCR sub-cloning. KMT2A mutant devoid of Su(var)3-9, Enhancer-of-zeste and Trithorax (SET; KMT2AΔSET) and Transactivation domain (TAD; KMT2AΔ TAD) were generated by deletion PCR.

G. Shows qRT-PCR analysis of KMT2A transcript levels in control cells, KMT2A full length (2A-FL), KMT2AΔSET (2AΔSET), and KMT2AΔTAD (2AΔTAD) cells. Error bars denote SD. *P ≤ 0.05, ** P ≤ 0.005, ***P≤ 0.0005, ns: not significant P > 0.05 (two-tailed Students t test).

H. Schematic of KMT2F full-length construct and KMT2F mutant (KMT2FΔSET) are shown. siRNA resistant (SR) KMT2F construct was generated by PCR-based mutagenesis, resulting in silent mutations. Subsequently, a KMT2F mutant lacking the SET domain was generated by deletion PCR from the FL SR construct.

I. qRT-PCR analysis of KMT2F transcript levels in control cells, KMT2F full length (2F-FL), and KMT2FΔSET (2FΔSET) cells. Error bars denote SD. *$P \leq 0.05$, ** $P \leq 0.005$, ***$P \leq 0.0005$, ns: not significant $P > 0.05$ (two-tailed Students t test). The underlying raw data pertaining to D-E & G-I can be found in S1 Data, while the uncropped blots for A-C are shown in the S1 Raw Images.

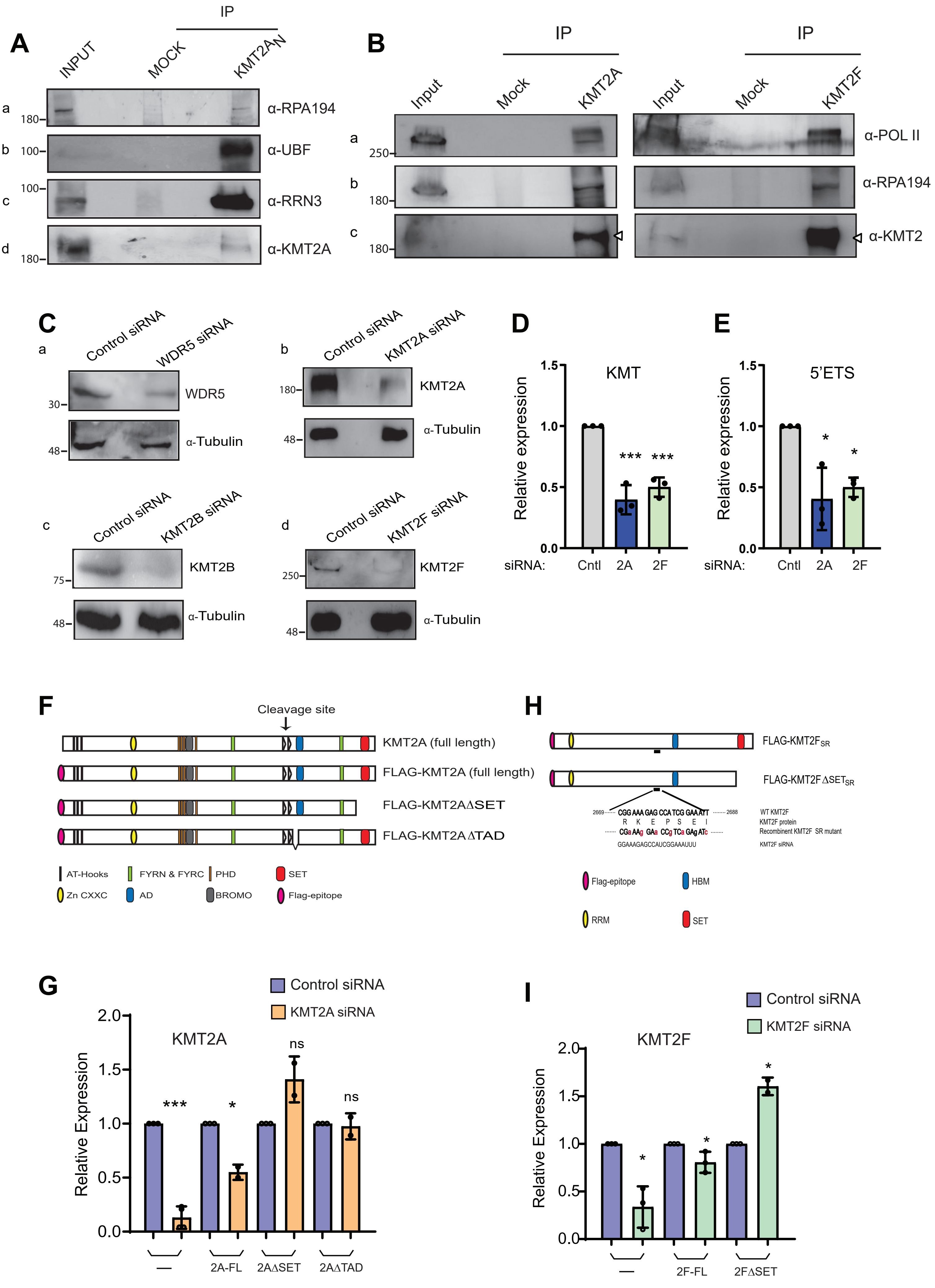

Lone Supplementary Figure 6
