# Supplementary material for: The KMT2F histone methyltransferase interacts with the RNA polymerase I machinery to promote ribosomal RNA transcription
Source: PLoS Biol. 2026 May 7;24(5):e3003785. doi: 10.1371/journal.pbio.3003785 (PMC13178980; doi:10.1371/journal.pbio.3003785)
Supplement: S7 Fig — (PDF) [file pbio.3003785.s007.pdf]

Supplementary Figure 7: Depletion of KMTs affects ribosomal DNA transcription in different cell lines.

A. Shows the FLAG (and DAPI) staining of stable cell lines of KMT2A and KMT2F, along with the U2OS control cells. KMT2A full-length, KMT2AΔSET, KMT2AΔTAD, KMT2F full-length and KMT2FΔSET expressing cells were stained with the FLAG antibody using paraformaldehyde fixation to visualize their nuclear localization indicating the expression of these exogenous proteins.

B. Representative autoradiogram displaying the 32P orthophosphate-labeled ribosomal RNA (45S and 32S) following KMT2A loss in various KMT2A mutant cell lines. U-2OS cells were treated with either control or KMT2A siRNA. Likewise, KMT2A F.L, KMT2AΔSET and KMT2AΔTAD cells received the same treatments. The total RNA extracted was analyzed on an agarose gel and visualized through autoradiography (upper panel). Additionally, 28S RNA levels were assessed using ethidium bromide staining on a formaldehyde agarose gel (lower panel).

C. Representative autoradiogram showing the 32P orthophosphate-labeled ribosomal RNA (45S and 32S) after KMT2F loss in various KMT2F mutant cell lines. U-2OS cells were treated with either control or KMT2F siRNA. Similarly, KMT2F F.L and KMT2FΔSET cells received the same treatments and were subjected to RNA extraction. The extracted total RNA was analyzed on an agarose gel and visualized via autoradiography (upper panel). Additionally, 28S RNA levels were evaluated using ethidium bromide staining on a formaldehyde agarose gel (lower panel).

D. Represents the autoradiogram depicting the 32P orthophosphate-labeled ribosomal RNA (45S and 32S) after the loss of different KMT2 members (KMT2A, KMT2F, KMT2B, KMT2C) and WDR5.

E. The effect of KMT2A loss on rRNA transcription in different cell lines is shown. HeLa, MCF7 and IMR90-tert cells were treated with KMT2A siRNA or control siRNA. Results here show densitometry quantification of 32P incorporation into 45S ribosomal RNA levels upon KMT2A loss. Each value

represents the result of two independent experiments, with standard deviation indicated by error bars. Statistical significance was assessed using a two-tailed Student's t test: $*P \leq 0.05$, $**P \leq 0.005$.

F. Effect of KMT2F loss on ribosomal transcript levels in HeLa and MCF7 cells. Cells were treated with KMT2F or Control siRNA and subjected to densitometry quantification of 32P incorporation into 45S ribosomal RNA levels. The relative intensity was measured by normalizing 45S levels with 28S rRNA loading control. Each value is an outcome of two independent experiments. S.D is represented by error bars. The statistical significance was calculated by two-tailed student t test $**P \leq 0.005$. The uncropped gel images from B-D can be found in the S1 Raw Images, while the underlying values for E-F can be found in S1 Data.

G. IFS of endogenous KMTB is shown in U-2OS cells. Cells were co-stained with KMT2B and B23 antibodies (DAPI, blue). Scale bar, 10 µm.

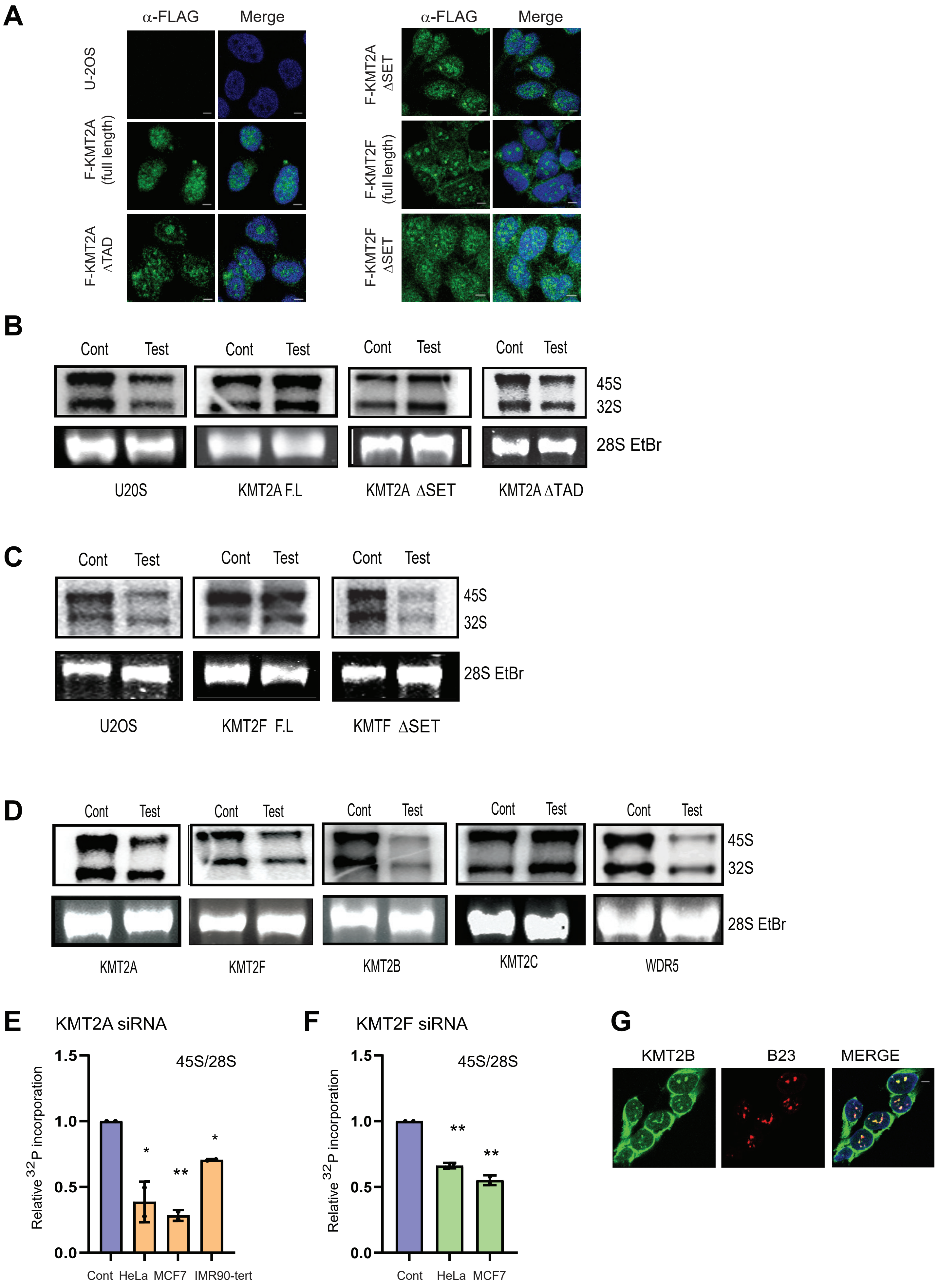

Lone Supplementary Figure 7
