# Supplementary material for: The KMT2F histone methyltransferase interacts with the RNA polymerase I machinery to promote ribosomal RNA transcription
Source: PLoS Biol. 2026 May 7;24(5):e3003785. doi: 10.1371/journal.pbio.3003785 (PMC13178980; doi:10.1371/journal.pbio.3003785)
Supplement: S8 Fig — (PDF) [file pbio.3003785.s008.pdf]

Supplementary Figure. 8. KMT2F deposits H3K4me3 marks to regulate the epigenetic state of human rDNA.

A-C. Represents ChIP analysis of KMT2F (**A**), H3K4Me2 (**B**), H3K4Me3 (**C**) in KMT2F shRNA KD. RAD18 and CD4 were used as positive and negative control primers. (**A-C**) Experiments were performed two or more times as biological replicates. Error bars represent SD. $*P \leq 0.05$, $** P \leq 0.005$, , ns: not significant $P > 0.05$ (two- tailed Students t test)

D. Represents immunoblots and quantifications of H3K4me2, H3K4me3 and H3 in KMT2F control and knockdown conditions. The blot was sequentially stripped and re-probed to visualize the levels of these modifications on the same blot. For quantifications, H3K4me2 and H3K4me3 levels in control and knockdown conditions were normalized to their respective H3 levels. Error bars represent SD. $*P \leq 0.05$, ns: not significant $P > 0.05$ (two- tailed Students t test)**.** The uncropped blots can be found in the S1 Raw Images.

E. Represents the ChIP analysis of KMT2C binding on PGR (Positive control) and SYN II (Negative control) regions of the genome. Error bars represent the SD of three biological replicates. Significance of KMT2C binding on PGR was calculated with respect to SYN II using students t test. $*P \leq 0.05$ (two- tailed Students t test).

F. Represents ChIP analysis of KMT2C on rDNA. Experiments were performed three times as biological replicates. Error bars represent SD. Significance was calculated with respect to the negative control primer binding for KMT2C (SYNII). All the rDNA primers showed non-significant binding on the rDNA (two-way ANOVA with Šídák multiple comparison test).

G. Shows the binding of KMT2C on the PGR and SYNII in KMT2C control and knockdown conditions. Results represent three independent biological replicates. Significance was calculated between the control and knockdown conditions for each primer pair using the two- tailed Students t test . $*P \leq 0.05$, ns: not significant $P > 0.05$.

H, I. Depicts the ChIP analysis of H3K4me2 (H) and H3K4me3 (I) in control & KMT2C knockdown conditions. Results represent three independent biological replicates. Error bars represent SD. Significance was calculated between the control and knockdown conditions for each primer pair using the two-way ANOVA with Šídák multiple comparison test, where all comparisons yielded non-significant changes. The underlying raw data pertaining to A-I can be found in S1 Data.

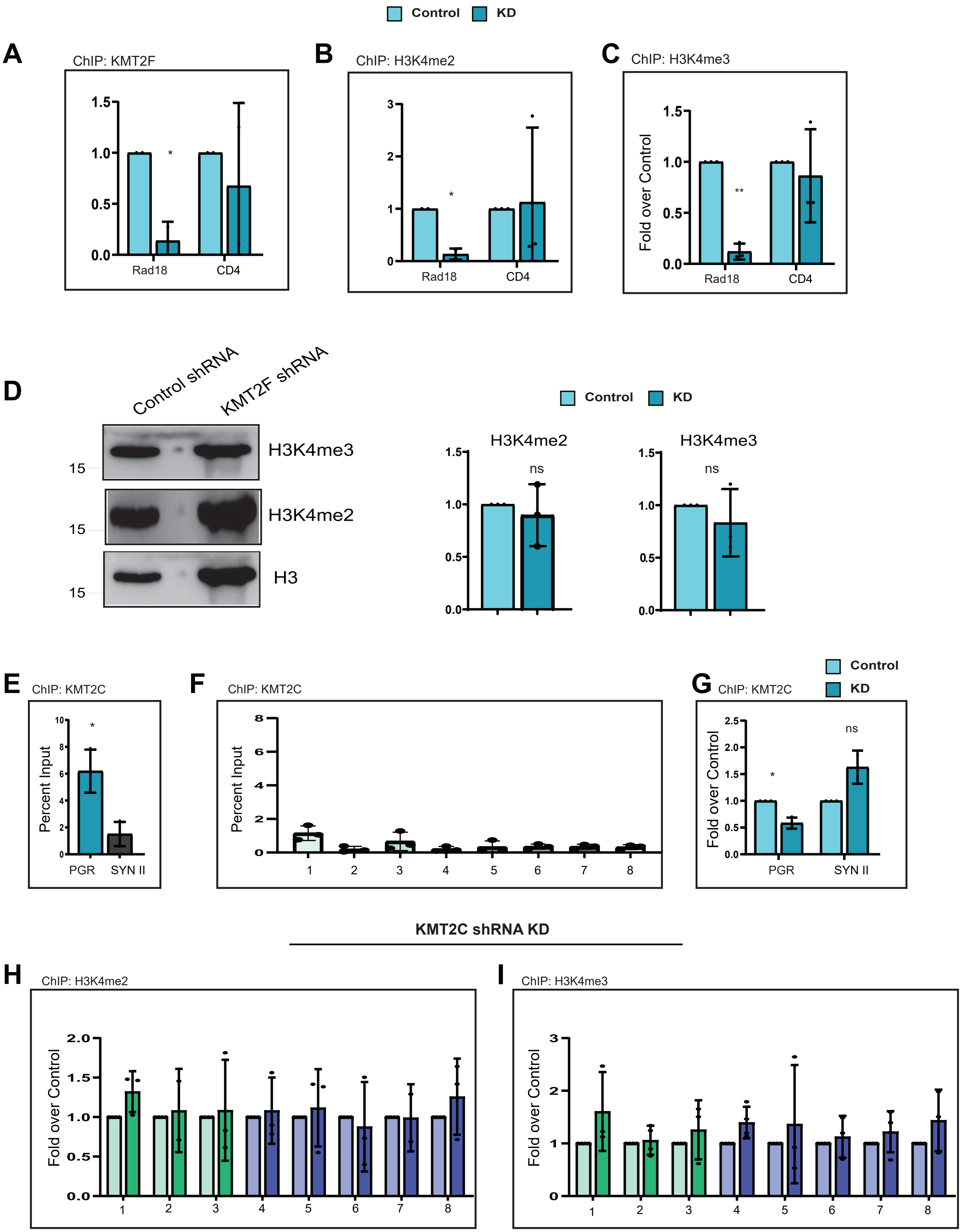

Lone Supplementary Figure 8
