# Supplementary material for: The KMT2F histone methyltransferase interacts with the RNA polymerase I machinery to promote ribosomal RNA transcription
Source: PLoS Biol. 2026 May 7;24(5):e3003785. doi: 10.1371/journal.pbio.3003785 (PMC13178980; doi:10.1371/journal.pbio.3003785)
Supplement: S9 Fig — (PDF) [file pbio.3003785.s009.pdf]

Supplementary Figure. 9. PIC formation under KMT2C depletion conditions

A-B. ChIP analysis of H3K9ac (A) & H3K9me3 (B) in KMT2F shRNA KD. RAD18 and CD4 were used as positive and negative control primers. Error bars indicate standard deviation (SD). Significance levels are as follows: *$P \leq 0.05$ (two-tailed Student's t test).

C,D. Shows the ChIP analysis of RNA Pol I (RPA194) (C) & UBF (D) in control & KMT2C knockdown conditions. Results represent three independent biological replicates. Error bars represent SD. Significance was calculated between the control & knockdown conditions for each primer pair using the two-way ANOVA with Šídák multiple comparison test. All the primer sets were showing the non-significant changes between the control and knockdown conditions. The underlying raw data pertaining to A-D can be found in S1 Data.

E. A schematic of the full-length KMT2F construct, along with its various domains, was used to PCR amplify the RRM domain fragment spanning amino acids 84 to 172. This fragment was then cloned into a vector containing the SFB tag. Ectopically expressed SFB-RRM and SFB were subjected to S-protein pulldowns. The RRM pull-down was detected using anti-FLAG antibody, and the immunoblot was probed with various antibodies (RPA194, RRN3, and RPA-49) to assess the interaction of RRM with different components of the pre-initiation complex. SFB, used as a mock control, was also detected with the anti-FLAG antibody. The uncropped blots for E can be found in the S1 Raw Images.

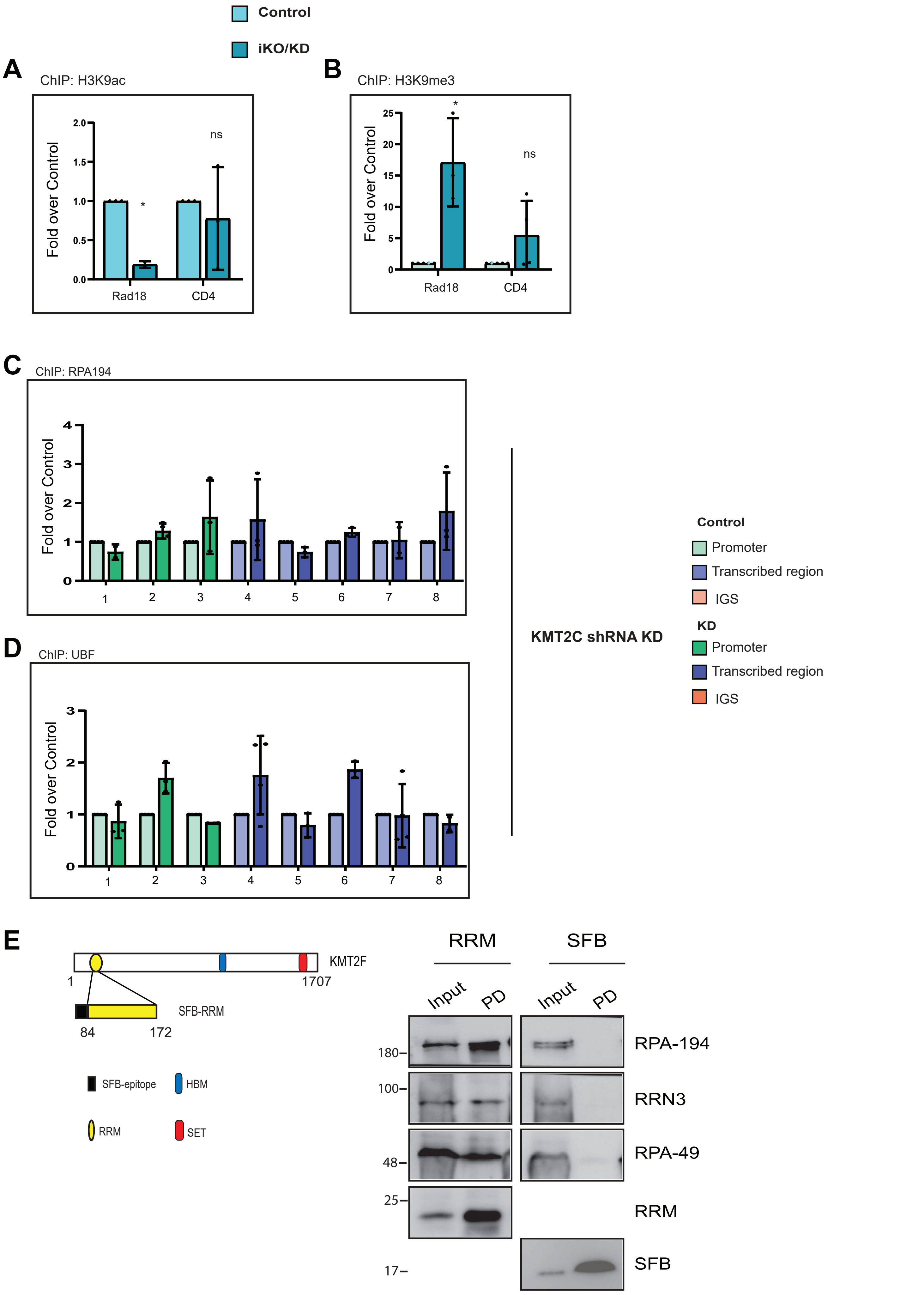

Lone Supplementary Figure 9
