# Supplementary material for: The KMT2F histone methyltransferase interacts with the RNA polymerase I machinery to promote ribosomal RNA transcription
Source: PLoS Biol. 2026 May 7;24(5):e3003785. doi: 10.1371/journal.pbio.3003785 (PMC13178980; doi:10.1371/journal.pbio.3003785)
Supplement: S1 Table — References for each primer set are also provided. (PDF) [file pbio.3003785.s010.pdf]

Primer no.	Position	Primer Sequence (Human rDNA)	Source
1	-813 / -707	F ACATAAACCTGCACGCCAGA<br>R CTAGGCAGAGCTCCGGAAA	This study
2	-410 / -272	F GATCCTTTCTGGCGAGTCC<br>R GGAGCCGGAAGCATTTTC	Zenter et al; 2011
3	-156 / +43	F GTGTGTGGCTGCGATGGT<br>R CCAACCTCTCCGACGACAG	Zenter et al; 2011
4	+1146 / +1305	F GGTCGTGTGTGGGTTGACTT<br>R GCGGTACGAGGAAACACCT	Zenter et al; 2011
5	+3990 / +4092	F CGACGACCCATTCGAACGTCT<br>R CTCTCCGGAATCGAACCCTGA	Zenter et al; 2011
6	+6697 / +6775	F GCAGGACACATTGATCATCG<br>R GACGCTCAGACAGGCGTAG	Zenter et al; 2011
7	+8256 / +8344	F GCTAAATACCGGCACGAGAC<br>R TTCACGCCCTCTTGAACTCT	Zenter et al; 2011
8	+12855 / +12970	F ACCTGGCGCTAAACCATTCGT<br>R GGACAAACCCTTGTGTCGAGG	Zenter et al; 2011
9	+18449 / +18591	F TGGTGGGATTGGTCTCTCTC<br>R CAGCCTGCGTACTGTGAAAA	Zenter et al; 2011
10	+24,028 / +24116	F CCCGCGCACATAATAACTAA<br>R AAATCACTCCTCACGGGAAC	This study
11	+28163 / +28298	F CACTACCCACGTCCCTTCAC	Zenter et al; 2011

		R GAGAGAAGACGGAGGCACAC	
12	+28279 / +28456	F GTGTGCCTCCGTCTTCTCTC	Zenter et al; 2011
		R GTCAAGGGGCTATGCCATC	
13	+28328 / +28495	F ATTCTTGCCAGGCTGACATT	Zenter et al; 2011
		R AAGCCTCACAACTGCAGACC	
14	28475 / +28597	F GTCTGCAGTTGTGAGGCTTT	This study
		R CGGAGGGCGGAGAAACTAAA	
15	+30541 / +30640	F ACTGGCGAGTTGATTTCTGG	Zenter et al; 2011
		R CGAGACAGTCGAGGGAGAAG	
16	+37,995 / +38,09	F CTCACAGAGGAAGGGAGCAC	This study
		R AACAAGGGAGGGAGGAACTT	
17	+41907 / +42035 Or - 1092 / - 964	F CCGTGGGTTGTCTTCTGACT	Zenter et al; 2011
		R AAGCGAAACCGTGAGTCG	
18	+42012 / +42202	F GCTTCTCGACTCACGGTTTC	Zenter et al; 2011
		R GGAGCTCTGCCTAGCTCACA	
**Canonical Gene Primers for ChIP**			
HoxA9	Fw.	CTCCGCCGCTCTCATTCTCAG	Malik et.al; 2022
	Rev.	GCCAGAAGGGGTGACTGTCC	
Rad18	Fw.	ATGCGCAGTACAAGCCCTTA	Malik et.al; 2022
	Rev.	GCTCCAACACCACTCGAAAT	

PGR	Fw.	GCTCCAGCTAACTGATGGTCTG	Jozwik et al: 2016
	Rev.	TGGGCCTAGATTATTGAGTTCAGG	
SYNP II	Fw.	AGAAGTACATACTGTTGTTGTCCC	Lee et al: 2007
	Rev.	AGAAATACCTCTTCTAGGGAGGAA	
U2C	Fw.	TTTGCTCCCACTGCCGTC	Malik et.al; 2022
	Rev.	CTGAGTCTTTCGGTGCCC	
CD4	Fw.	TCTGCAGAAGGAACAAAGCA	Malik et.al; 2022
	Rev.	GGAAGGAAGCCGAGTCTGA	
**Transcriptional Primers**			
KMT2A	Fw.	GGAGCACACATTCCAGACCA	Malik et.al; 2023
	Rev.	TTTGGGTCACCTGAACTTCC	
KMT2F	Fw.	CGAATACGTGGGTCAGAACA	Malik et.al; 2023
	Rev.	TGCAGCAGTGGTTGATGAAT	
KMT2B	Fw.	AGCCGTGTGAGGATGAAAAC	Malik et.al; 2023
	Rev.	ACCTGGGGAGGACCATCTT	
KMT2C	Fw.	CAGCACCACGAAAACAAAGA	Malik et.al; 2023
	Rev.	ACTCCACACAACGGTGATGA	
GAPDH	Fw.	CGAGATCCCTCCAAAATCAA	Malik et al; 2023
	Rev.	TTCACACCCATGACGAACAT	
WDR5	Fw.	TGCCTGAAGACGTACACT	This study
	Rev.	TTCTGTTGGGTGACAAGC	
3′	Fw.	GTCCCCTCGTCTGCTCCTCTC	This study
	Rev.	CAAGTCGACAACCACTG	

**shRNA sequences**			
KMT2F shRNA	shRNA#1	5’-AGCAAAAGGGACCCACCCC	Malik et al; 2023
	shRNA#2	5’GACAACAACGAATGAAATA	Deng, C. *et al 2013*
KMT2C	shRNA#1	5’CGCACCTTATAGTAAACAGTT	This study
shRNA	shRNA#2	5’GCTGGCCACGGCCTTATTAAA	This study
Scramble		5’-GCGCGATAGCGCTAATAATTT	Liu et al; 2007

Reference:

1. Deng, C. *et al.* USF1 and hSET1A Mediated Epigenetic Modifications Regulate Lineage Differentiation and HoxB4 Transcription. *PLoS Genet.* **9**, (2013).

2. Jozwik, K. M., Chernukhin, I., Serandour, A. A., Nagarajan, S., & Carroll, J. S. (2016). FOXA1 directs H3K4 monomethylation at enhancers via recruitment of the methyltransferase MLL3. Cell reports, 17(10), 2715-2723.

3. Lee, J.-H., & Skalnik, D. G. (2008). Wdr82 Is a C-Terminal Domain-Binding Protein That Recruits the Setd1A Histone H3-Lys4 Methyltransferase Complex to Transcription Start Sites of Transcribed Human Genes. *Molecular and Cellular Biology*, *28*(2), 609–618. https://doi.org/10.1128/mcb.01356-07

4. Liu, H., Cheng, E. H. Y. & Hsieh, J. J. D. Bimodal degradation of MLL by SCFSkp2 and APCCdc20 assures cell cycle execution: A critical regulatory circuit lost in leukemogenic MLL fusions. *Genes Dev.* **21**, 2385–2398 (2007).

5. Malik, K. K., Sridhara, S. C., Lone, K. A., Katariya, P. D., Pulimamidi, D., & Tyagi, S. (2023). MLL methyltransferases regulate H3K4 methylation to ensure CENP-A assembly at human centromeres. *PLOS Biology*, *21*(6), e3002161. https://doi.org/10.1371/journal.pbio.3002161

6. Zentner, G. E., Saiakhova, A., Manaenkov, P., Adams, M. D., & Scacheri, P. C. (2011). Integrative genomic analysis of human ribosomal DNA. *Nucleic Acids Research*, *39*(12), 4949–4960. https://doi.org/10.1093/nar/gkq1326
