# Supplementary material for: The KMT2F histone methyltransferase interacts with the RNA polymerase I machinery to promote ribosomal RNA transcription
Source: PLoS Biol. 2026 May 7;24(5):e3003785. doi: 10.1371/journal.pbio.3003785 (PMC13178980; doi:10.1371/journal.pbio.3003785)
Supplement: S1 Raw Images — A PDF file containing all uncropped Western blots or gel images used in this manuscript. (PDF) [file pbio.3003785.s012.pdf]

S1_Raw Images

Figure 3B

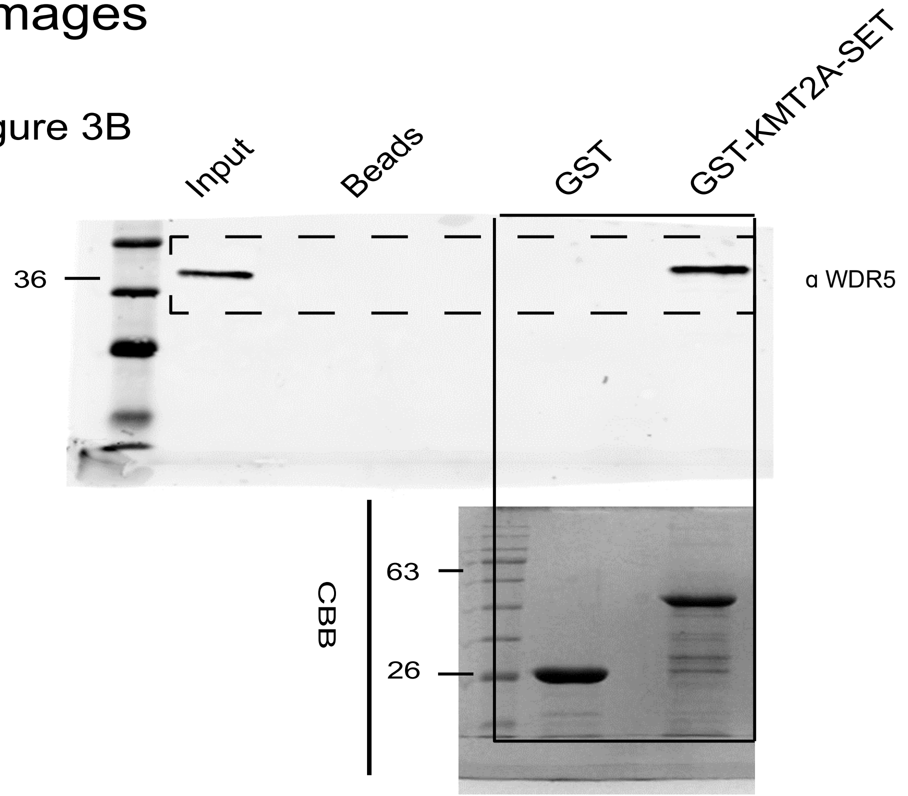

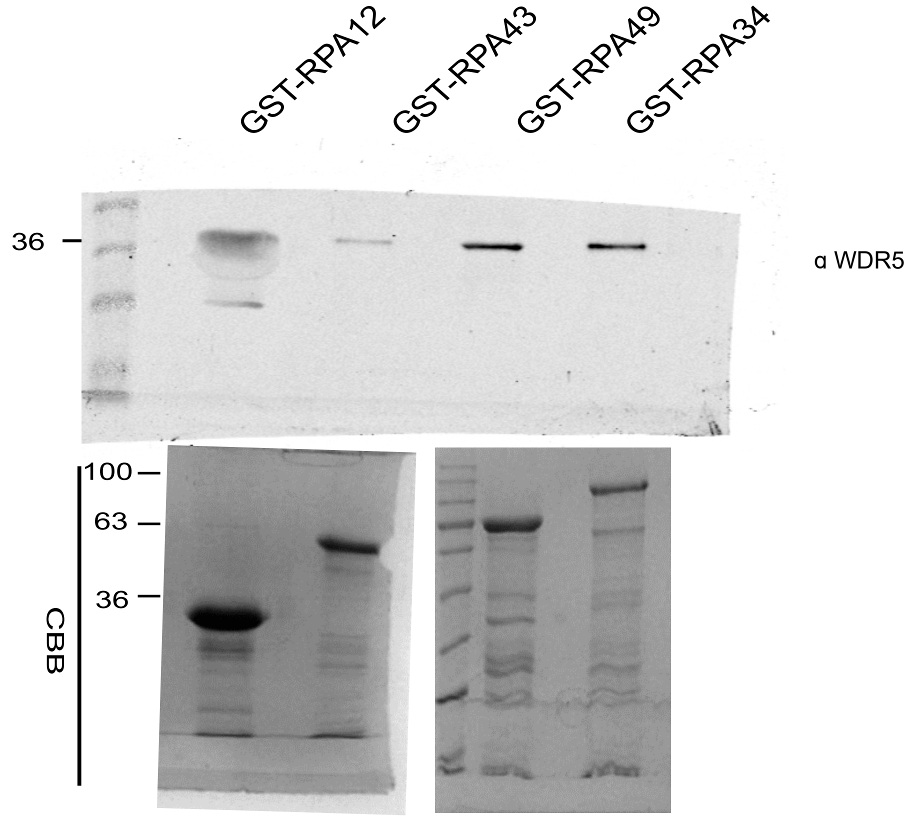

Figure 3C

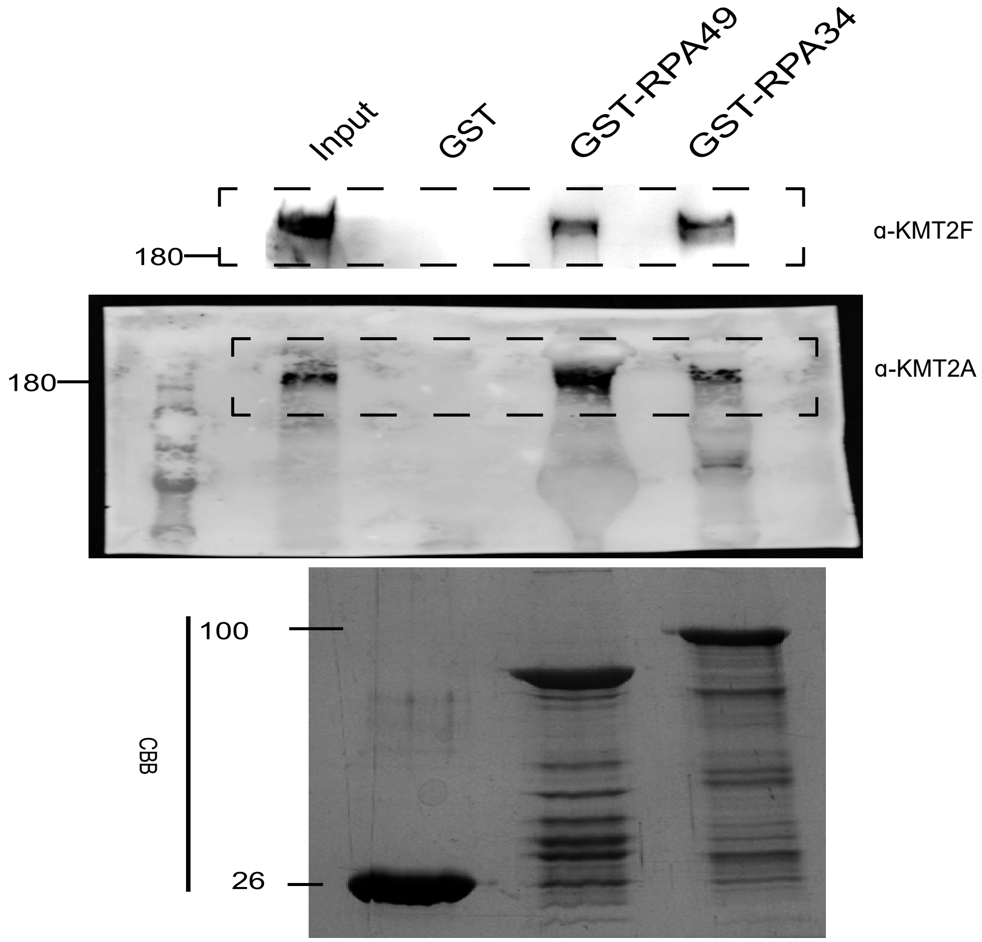

Input
GST
GST-RPA49
GST-RPA34
180
α-KMT2F
180
α-KMT2A
100
CBB
26

Figure 3D

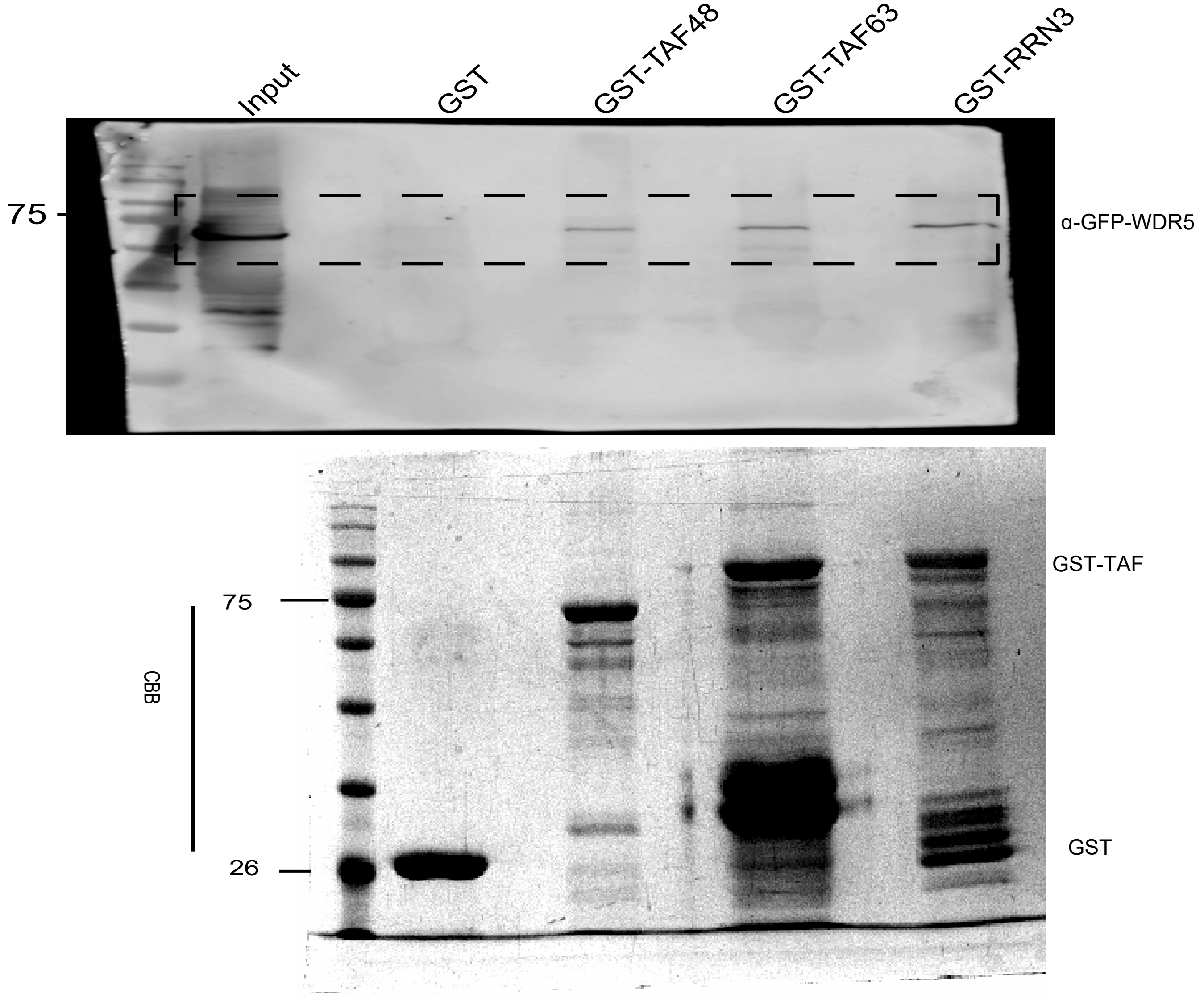

Input
GST
GST-TAF48
GST-TAF63
GST-RRN3
75
α-GFP-WDR5
75
26
CBB
GST-TAF
GST

Figure 3E

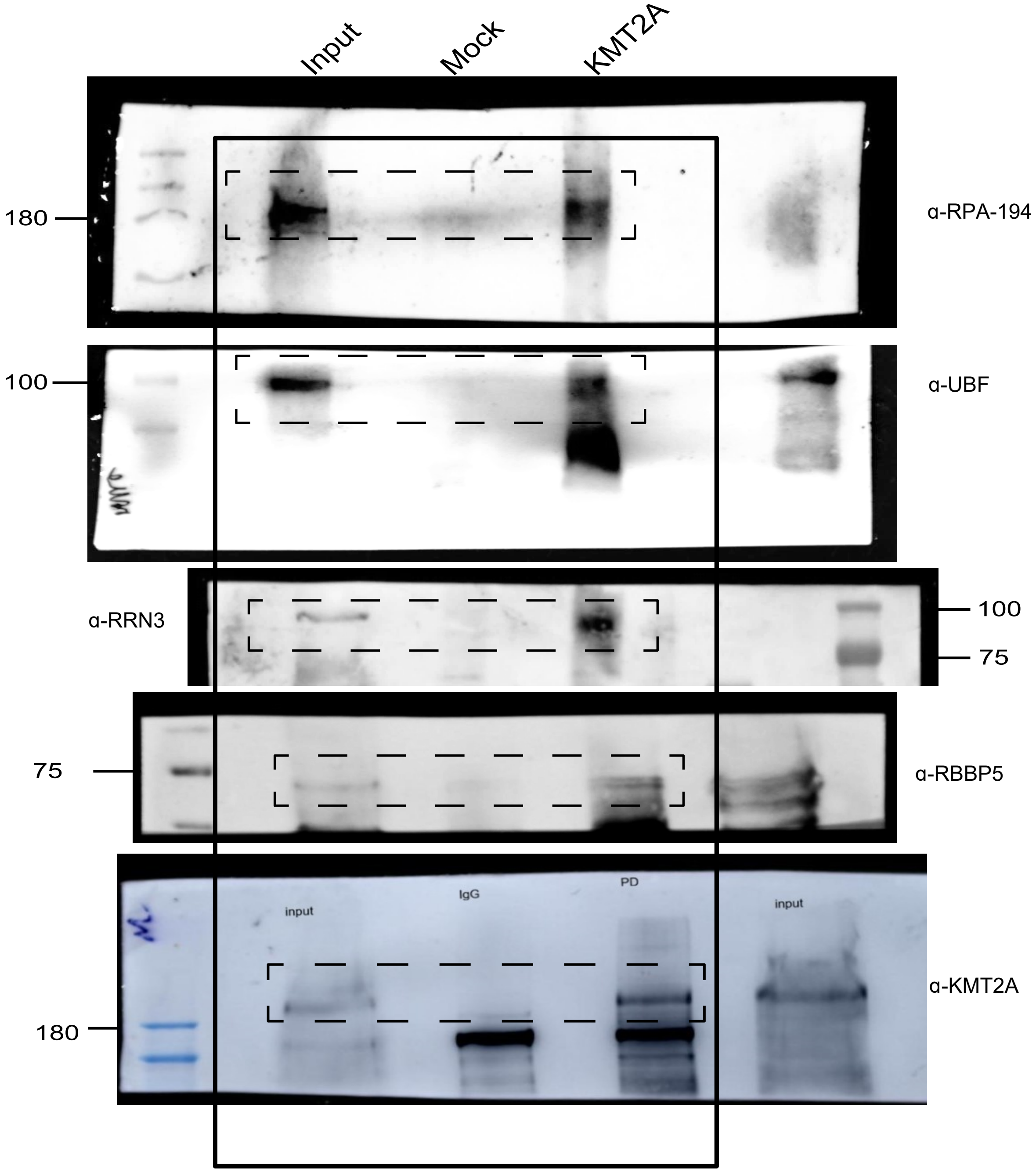
Input
Mock
KMT2A
180
α-RPA-194
100
α-UBF
α-RRN3
100
75
75
α-RBBP5
input
IgG
PD
input
α-KMT2A
180

Figure 3E

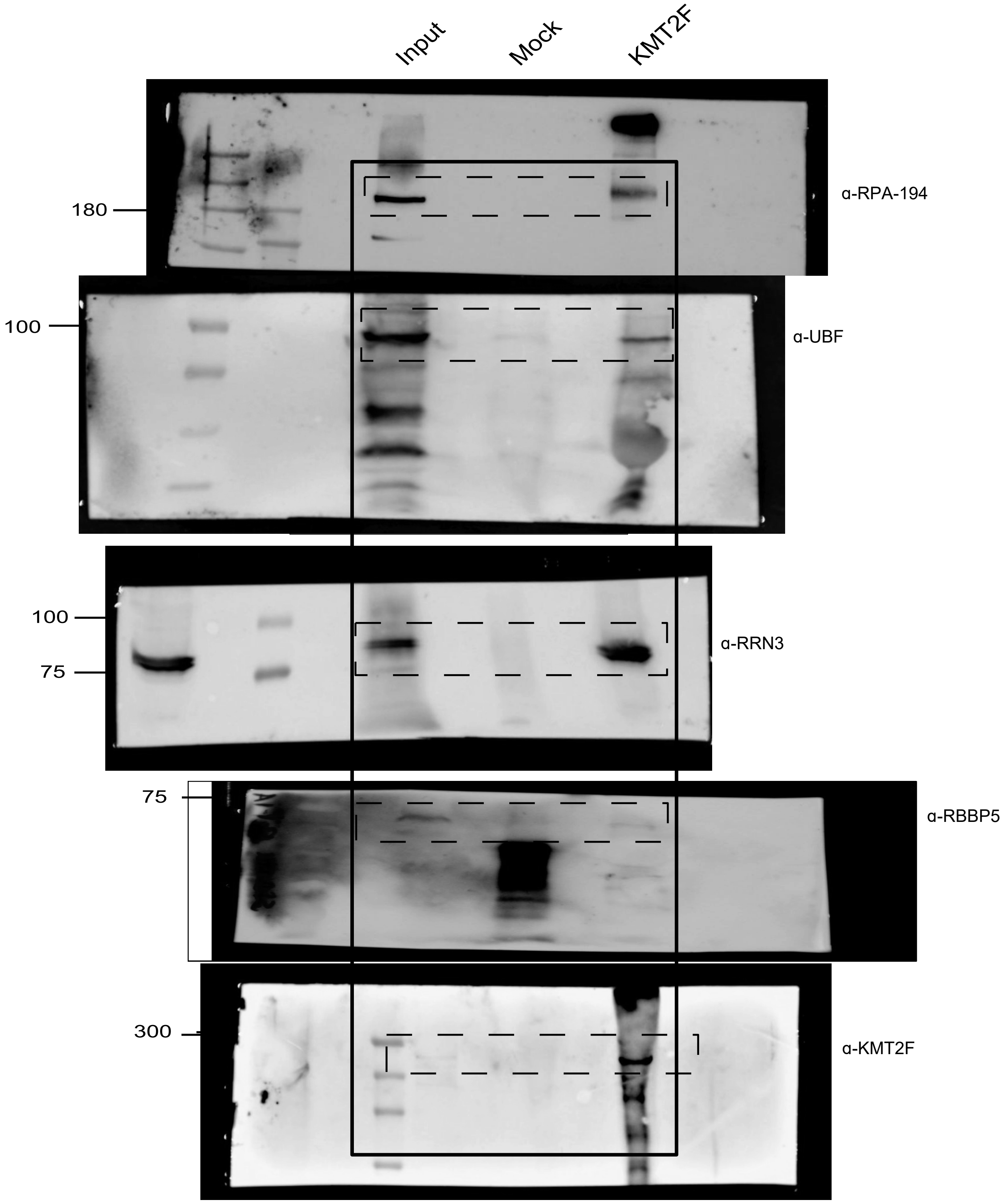
Input
Mock
KMT2F
180
α-RPA-194
100
α-UBF
100
75
α-RRN3
75
α-RBBP5
300
α-KMT2F

Figure 4E

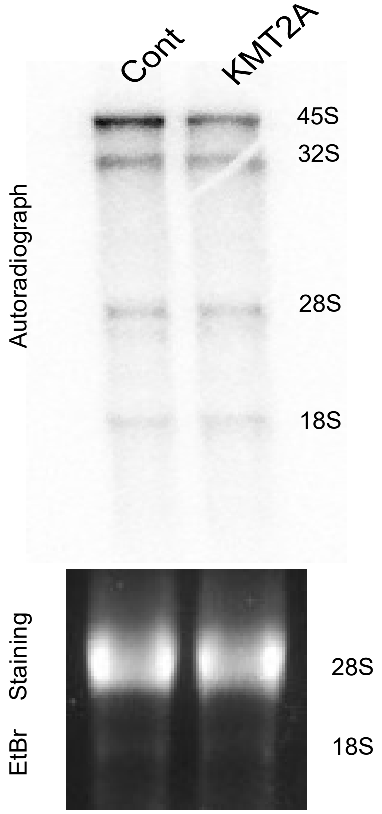

Figure 5A

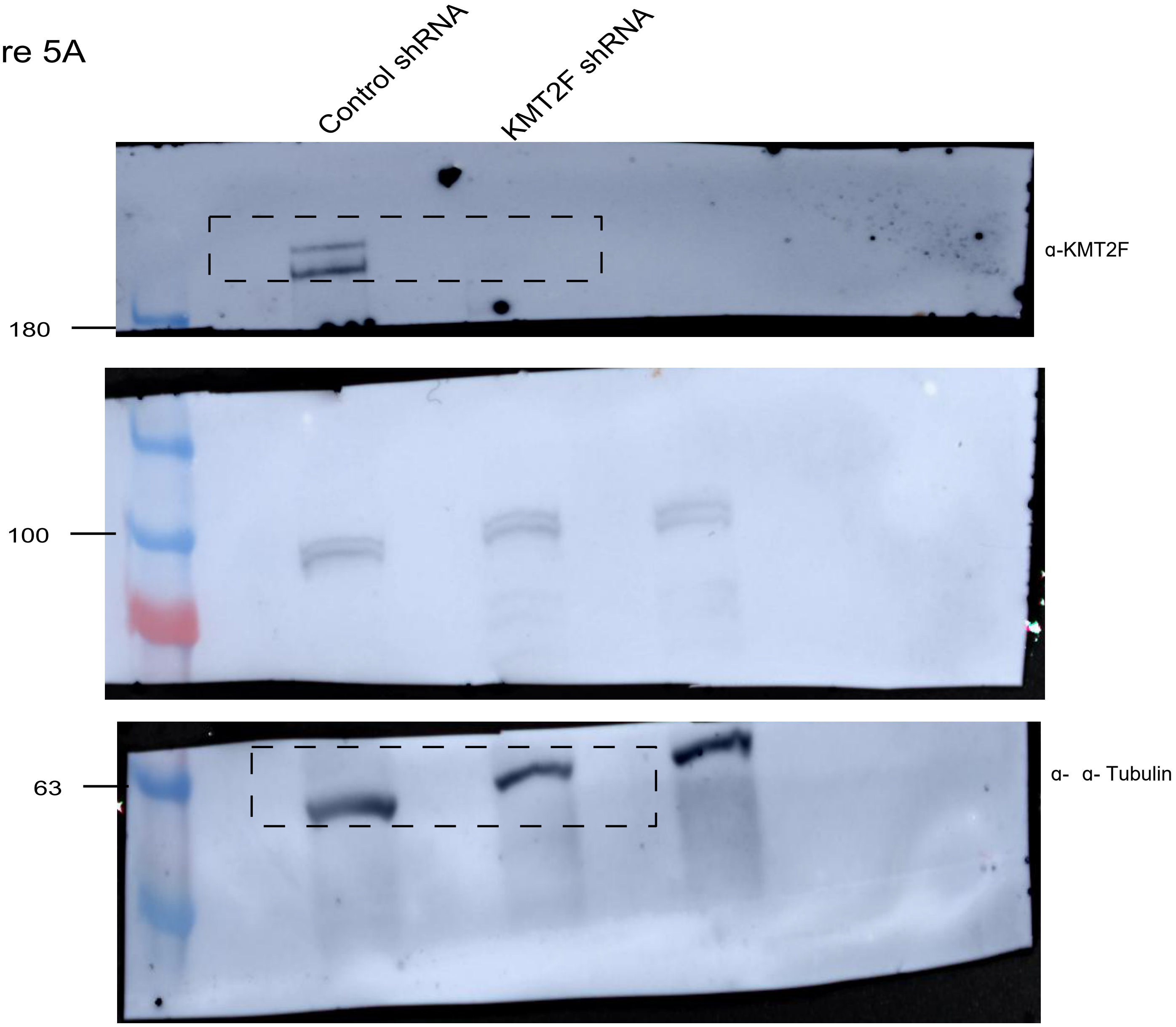

Figure 7A

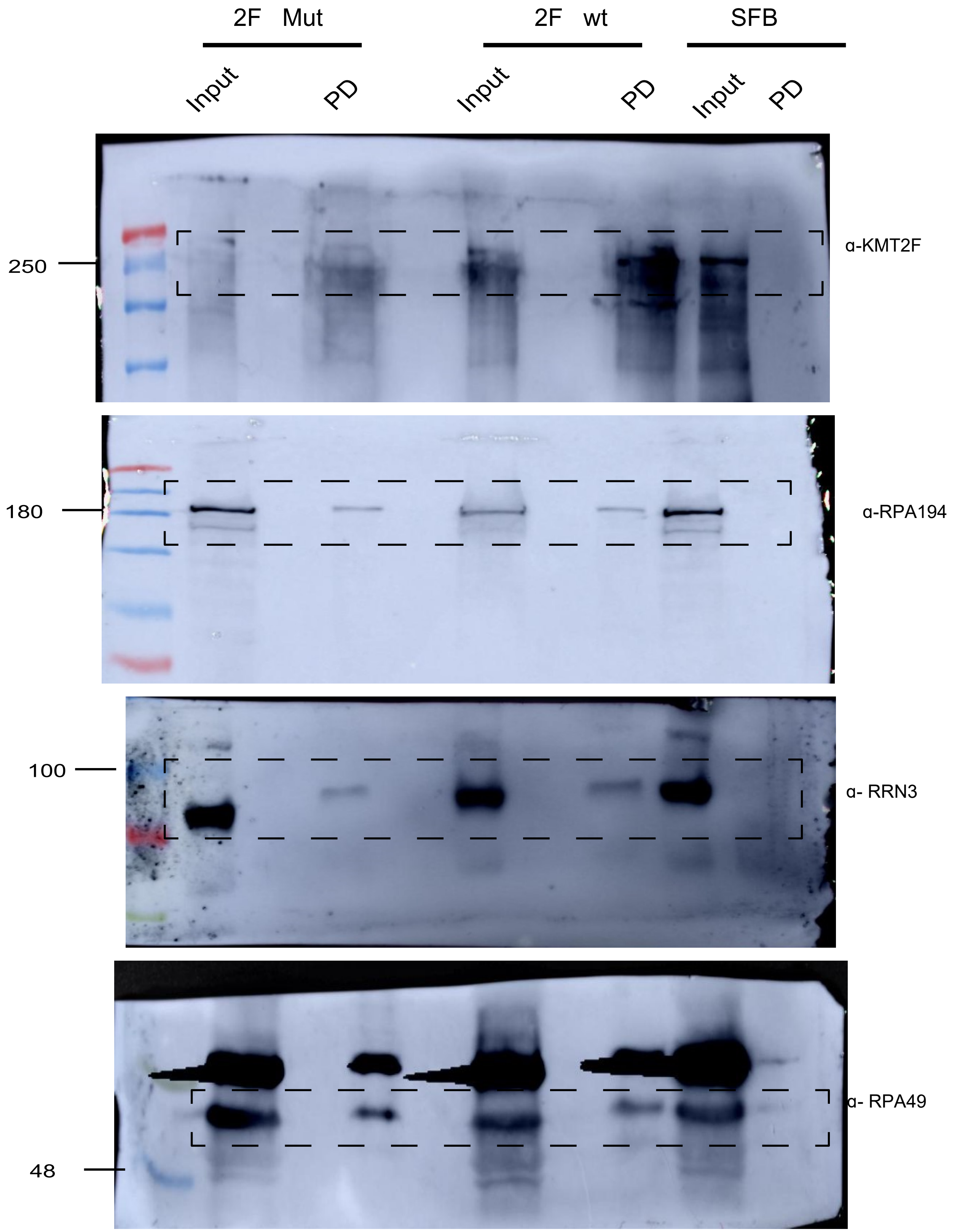
2F Mut
2F wt
SFB
Input
PD
Input
PD
Input
PD
250
α-KMT2F
180
α-RPA194
100
α- RRN3
α- RPA49
48

Figure S1

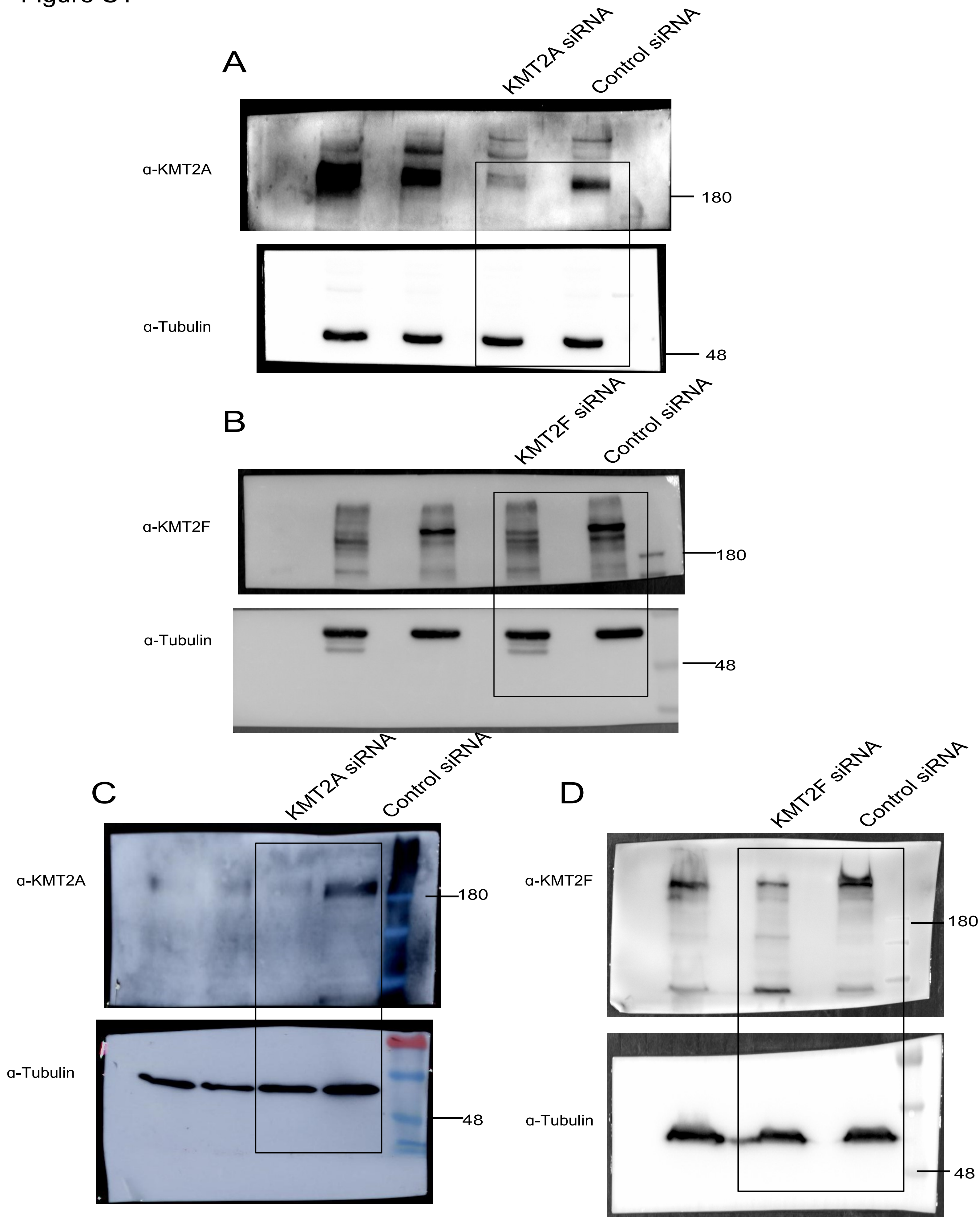
A
KMT2A siRNA
Control siRNA
α-KMT2A
180
α-Tubulin
48
B
KMT2F siRNA
Control siRNA
α-KMT2F
180
α-Tubulin
48
C
KMT2A siRNA
Control siRNA
α-KMT2A
180
α-Tubulin
48
D
KMT2F siRNA
Control siRNA
α-KMT2F
180
α-Tubulin
48

Figure S6A

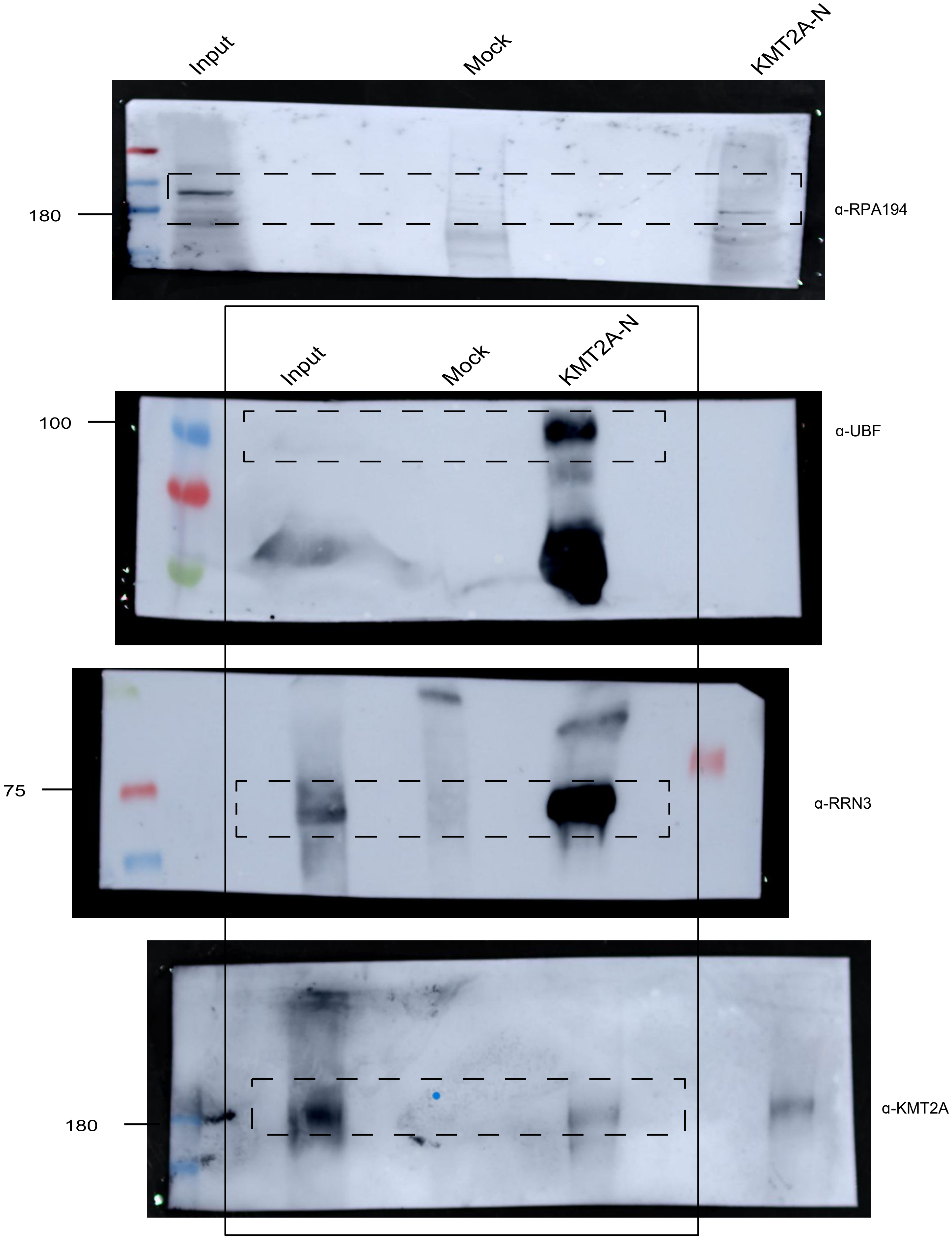
Input
Mock
KMT2A-N
180
α-RPA194
Input
Mock
KMT2A-N
100
α-UBF
75
α-RRN3
180
α-KMT2A

Figure S6
S6B

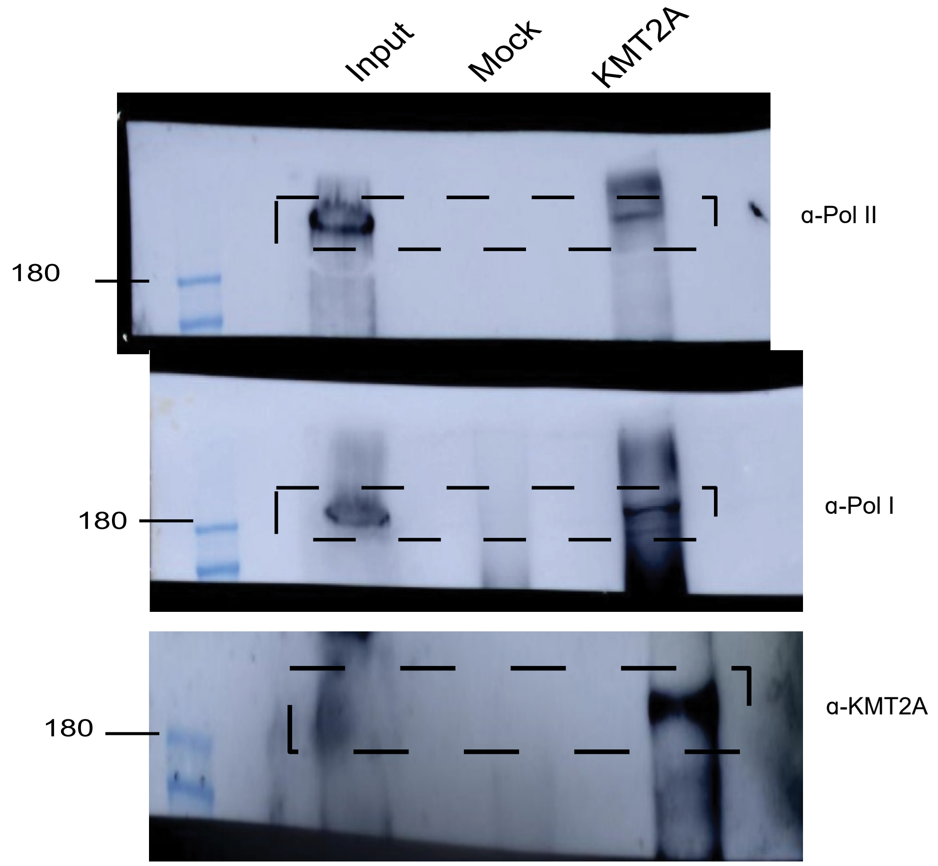

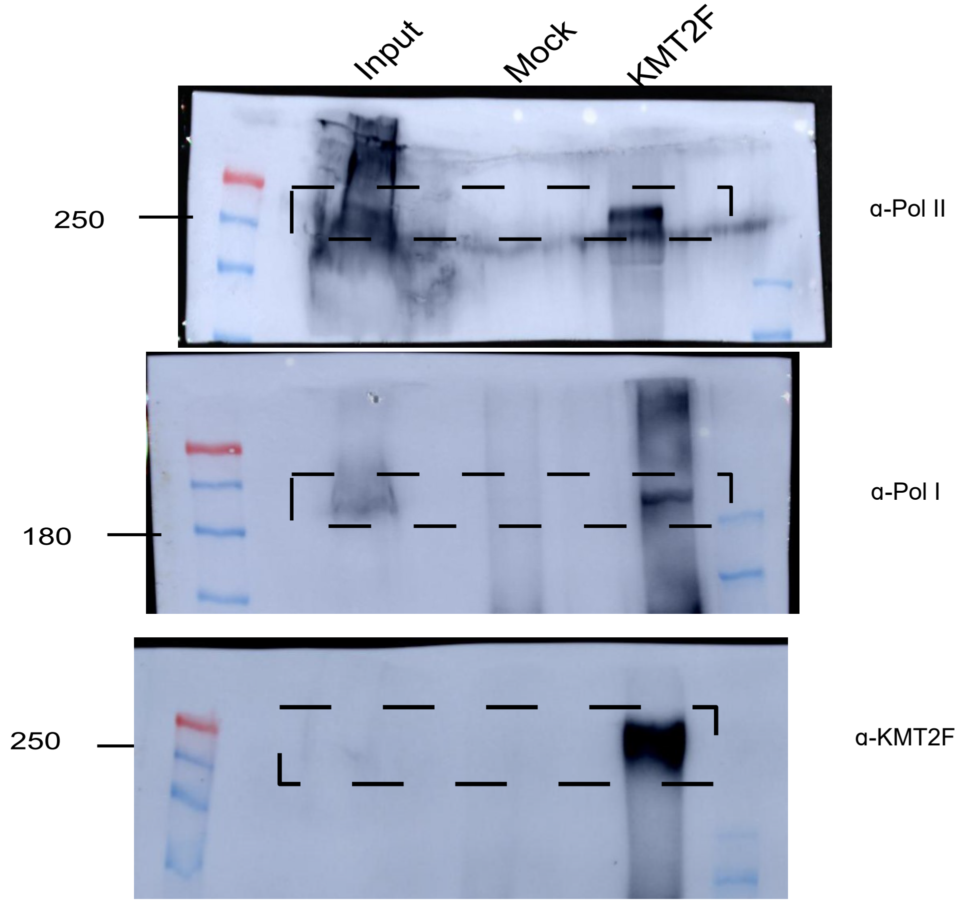

S6C

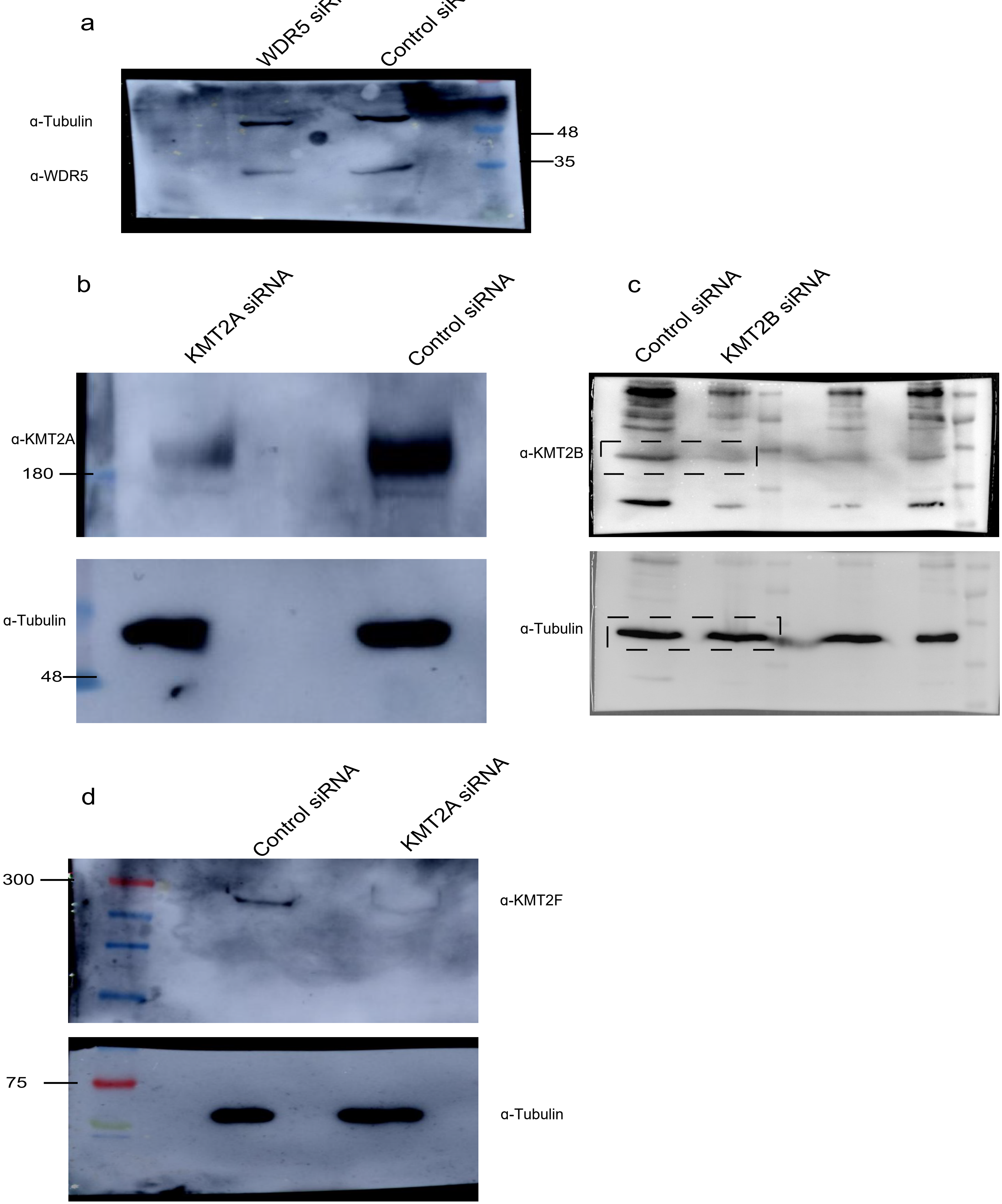
a
WDR5 siRNA
Control siRNA
α-Tubulin
α-WDR5
48
35
b
KMT2A siRNA
Control siRNA
α-KMT2A
180
α-Tubulin
48
c
Control siRNA
KMT2B siRNA
α-KMT2B
α-Tubulin
d
Control siRNA
KMT2A siRNA
300
α-KMT2F
75
α-Tubulin

Figure S7 B-C

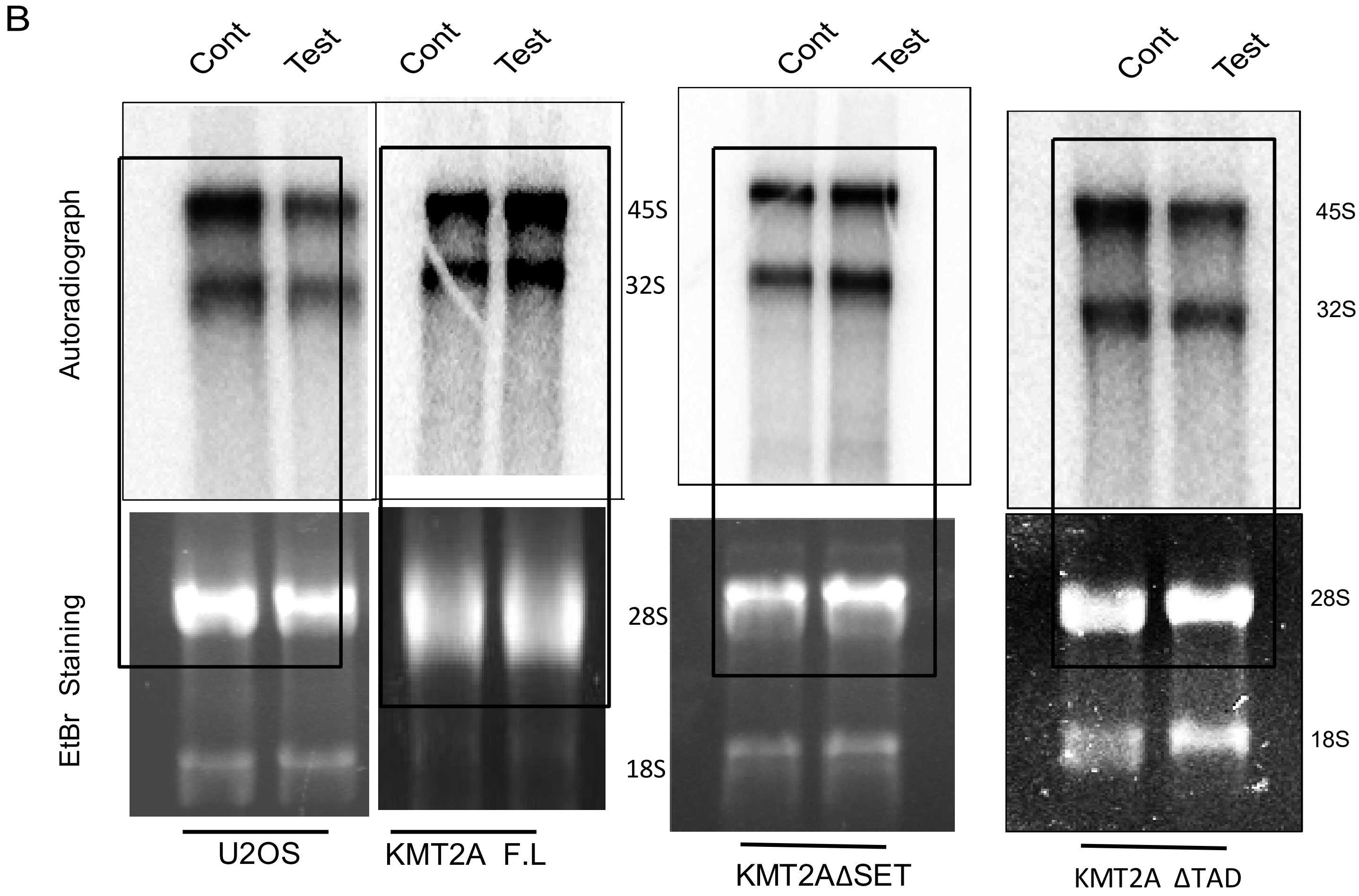
B
Cont
Test
Cont
Test
Cont
Test
Cont
Test
Autoradiograph
EtBr Staining
45S
32S
28S
18S
45S
32S
28S
18S
U2OS
KMT2A F.L
KMT2AΔSET
KMT2A ΔTAD

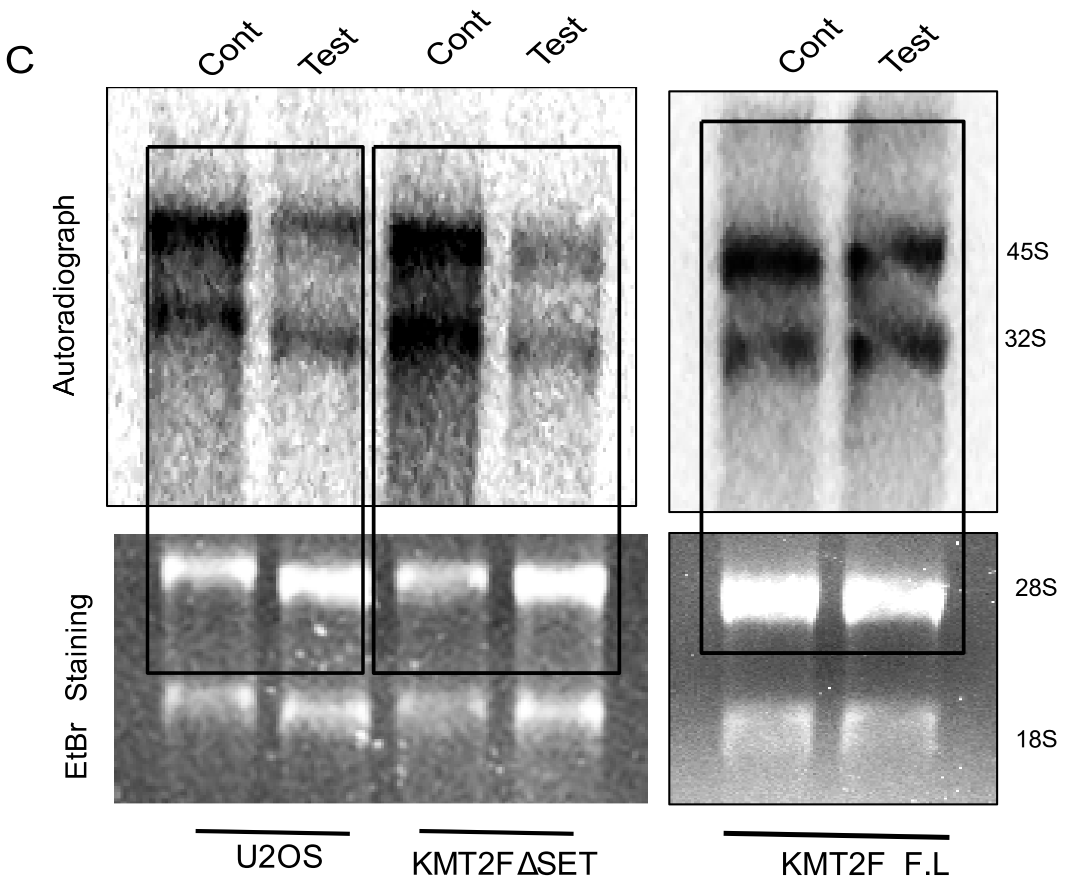
C
Cont
Test
Cont
Test
Cont
Test
Autoradiograph
EtBr Staining
45S
32S
28S
18S
U2OS
KMT2FΔSET
KMT2F F.L

Figure S7D

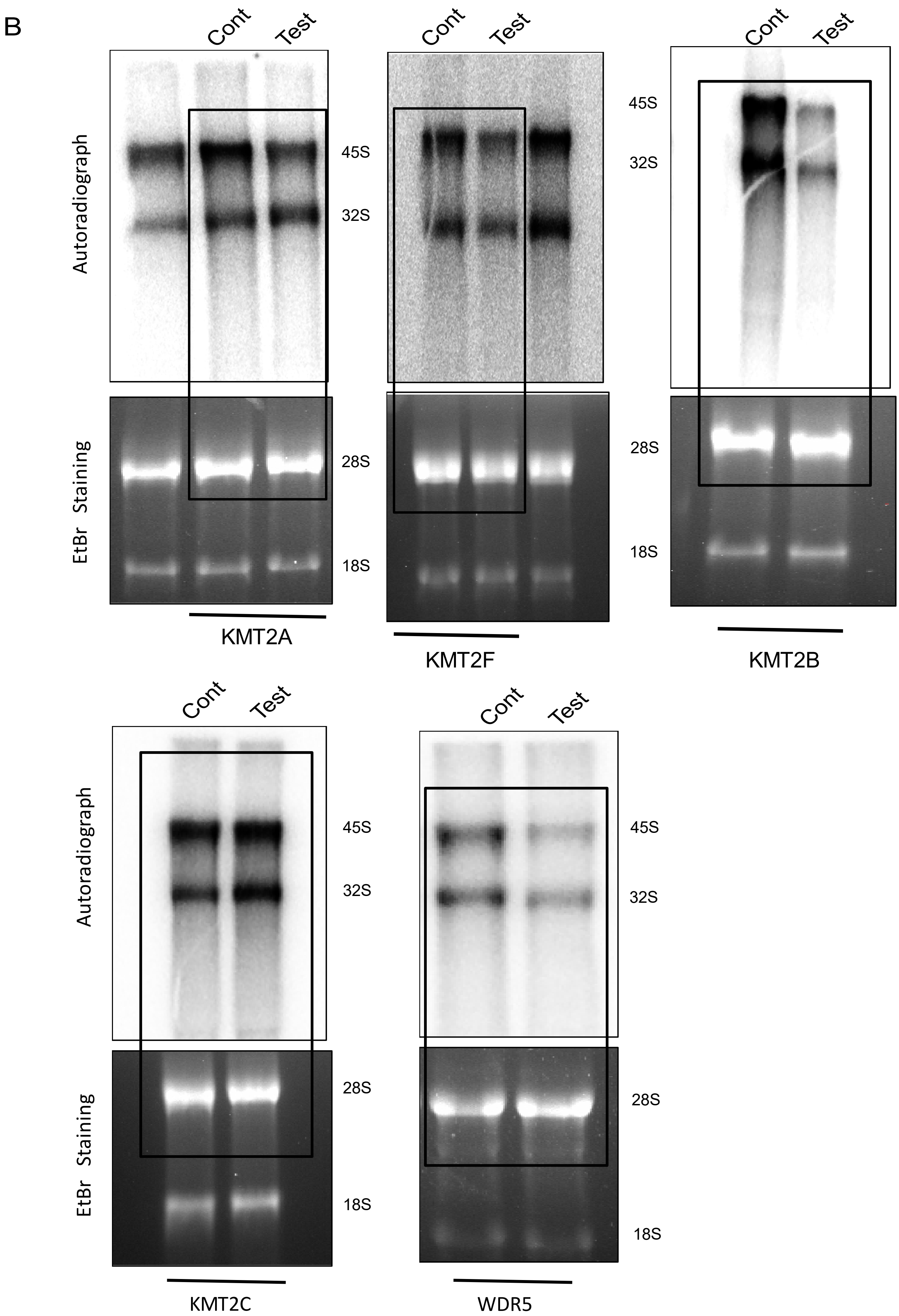
B
Cont
Test
Autoradiograph
EtBr Staining
45S
32S
28S
18S
KMT2A
KMT2F
KMT2B
KMT2C
WDR5

Figure S8D

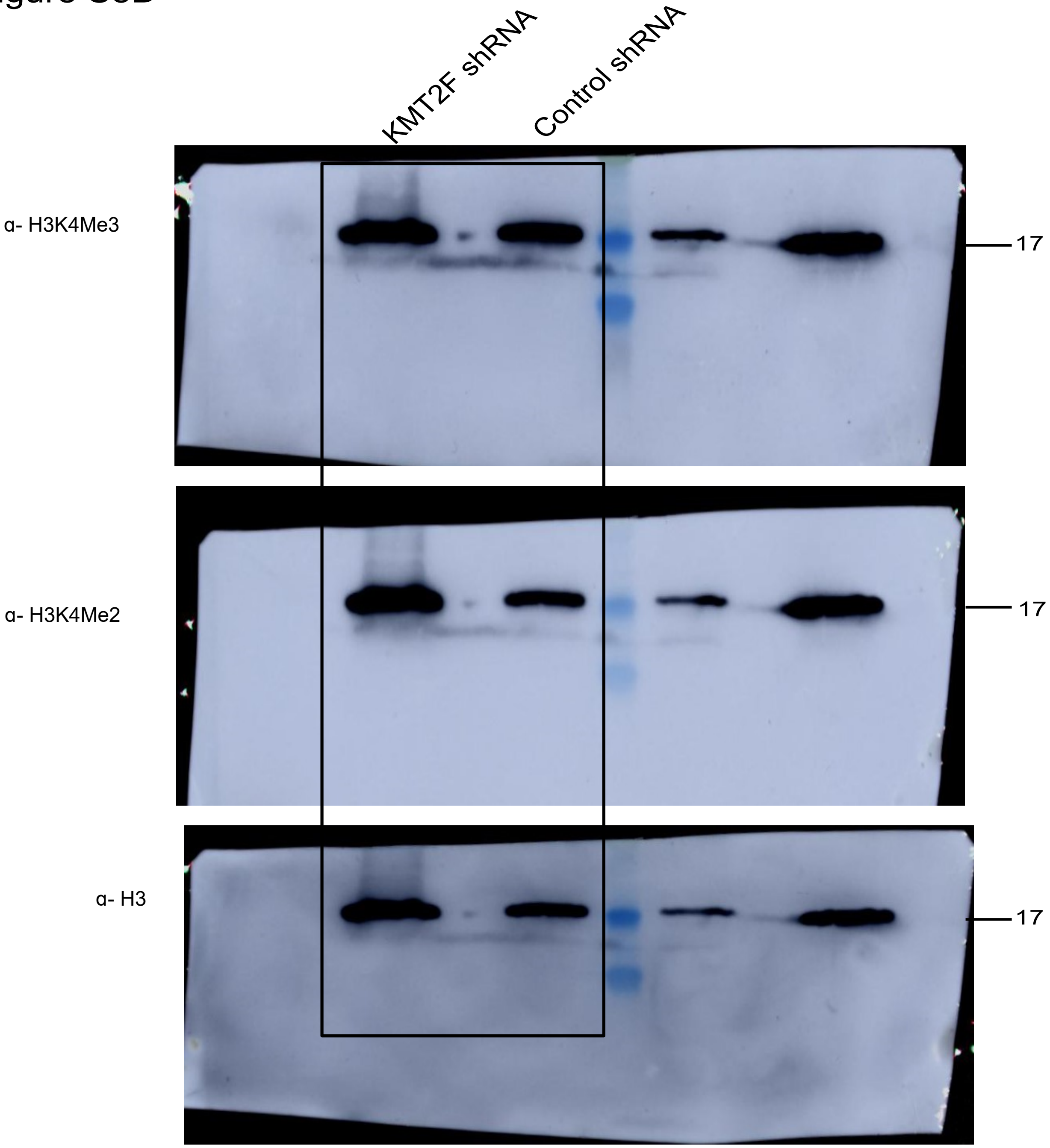

Figure S9E

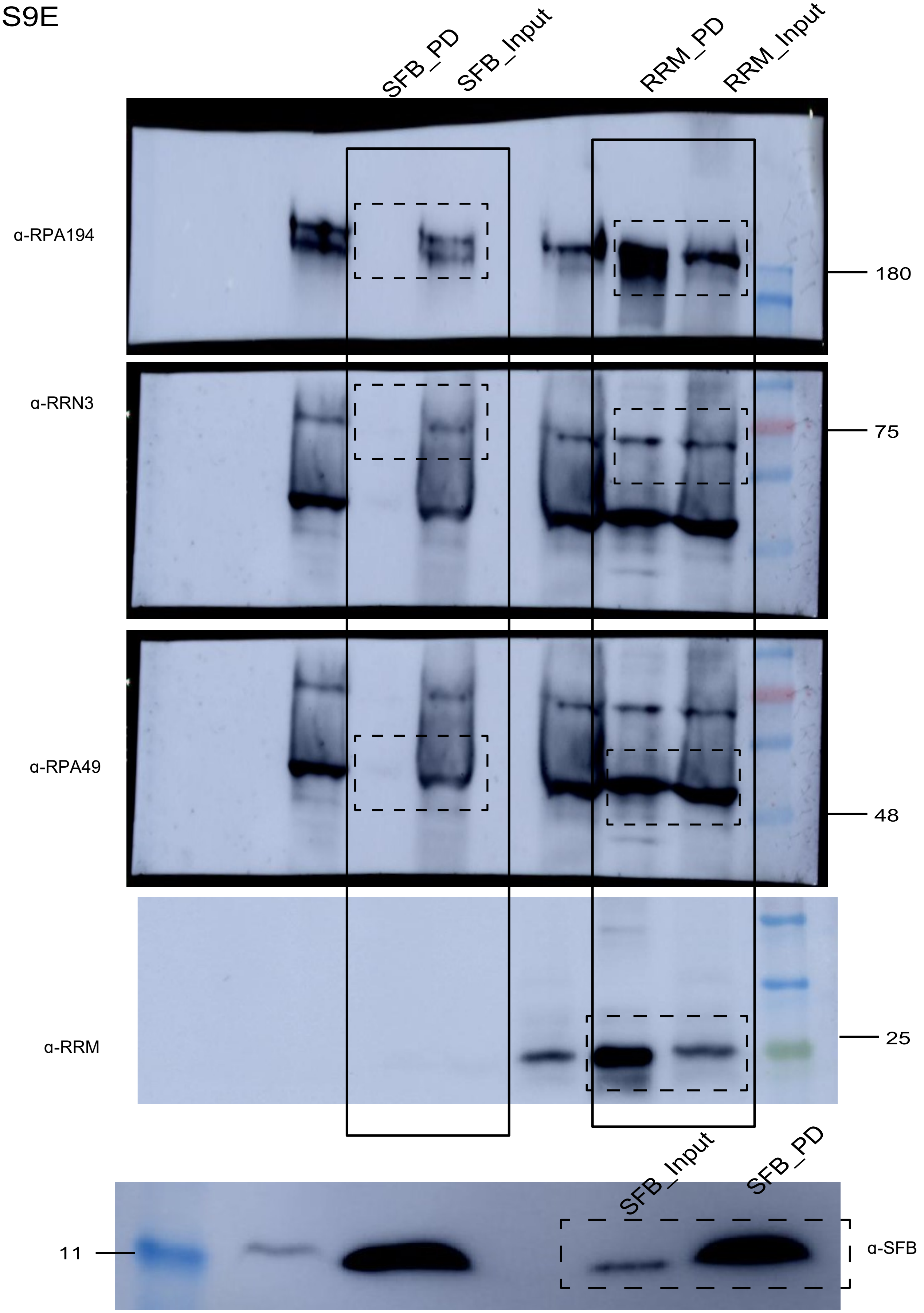
SFB_PD
SFB_Input
RRM_PD
RRM_Input
α-RPA194
180
α-RRN3
75
α-RPA49
48
α-RRM
25
SFB_Input
SFB_PD
11
α-SFB
