# Supplementary material for: The KMT2F histone methyltransferase interacts with the RNA polymerase I machinery to promote ribosomal RNA transcription
Source: PLoS Biol. 2026 May 7;24(5):e3003785. doi: 10.1371/journal.pbio.3003785 (PMC13178980; doi:10.1371/journal.pbio.3003785)
Supplement: Attachment — Submitted filename: Response to Reviewers.pdf [file pbio.3003785.s015.pdf]

Response to Reviewers Reviewer: they/them

We thank all the Reviewers for their positive reviews. We note that in general, all Reviewers find the study interesting, are satisfied with the quality of the experiments and conclusions drawn. In addition, they would like to see further experiments done to strengthen the current findings/conclusions.

Some of the key experiments we have addressed/ added in the manuscript are as follows:

1. Positive and negative controls for our ChIP-seq experiments
2. Genome-wide analysis of ChIP-seq data for KMT2A/2F and H3K4me2/me3.
3. Epigenome and PIC formation upon loss of KMT2C
4. Analyses of broad transcriptional reprogramming upon KMT2A and KMT2F knockdown.
5. RNA Pol II interaction with the KMTs as a positive control for co-IPs
6. Using same cell line for demonstrating various results.
7. Correlation of ChIP–qPCR and ChIP-seq datasets.

We feel that the results from the above experiments have strengthened our present claim considerably.

The data generated has led to the addition and reorganisation of figures as detailed below:

**Main Figures**		
**Old Figure No.**	**Now changed to**	**Description of changes made/ Newly added data**
Figure 1	Same as before	
Figure 2	Same as before	

Figure 3	Same as before	
Figure 4	Same as before	Shows transcript analysis of rDNA also rescue experiment for all KMT2 proteins
Figure 5	Same as before	
Figure 6	Same as before	
Figure 7	New	Co-IPs of KMT2F (wild type and mutant) with PIC formation (moved from previous S7).
	**Supplementary Figures**	
Supplemental Figure S1	Same	IFS of KMT2A and KMT2F in HEK293, validation by knock down with WB, and overexpression of GFP-KMT2A/F along with the Ds-Red B23<br>All images deconvoluted/replaced.
Supplemental Figure S2	Same	ChIP seq track with negative control
Supplemental Figure S3	Same	
	New Supplemental Figure S4	Primer read counts from ChIP-seq data
	New Supplemental Figure S5	Quantitive analysis of ChIP seq & RNA seq data
Supplemental Figure S4	New Supplemental Figure S6	Interaction of KMT2A & KMT2F with Pol II, siRNA knockdown blots of KMT2A,2B, 2C & WDR5. Transcript analysis for KMT2/5ETS in Hek293 cells,

Supplemental Figure S5	New Supplemental Figure S7	IFS with anti-FLAG antibody for verifying transgene expression.
Supplemental Figure S6	New Supplemental Figure S8	WB of histone modifications, KMT2C ChIP data, Epigenome ChIP in KMT2C knockdown
	New Supplemental Figure S9	PIC data in KMT2C KD S9

Details of all experiments performed to address their comments are given as a point-by-point response in the document.

Note on KMT2A

The Reviewers have asked us to evaluate epigenomics and PIC status on KMT2A knockdowns. In this context, we wish to provide some background.

We had analysed KMT2F and KMT2A together in an earlier version of the manuscript (posted on bioRxiv as Lone et al., 2024) that was submitted to previous journals. However, presenting these results simultaneously proved confusing to the Reviewers. Consequently, in the current submission to *PLOS Biology* (also available on bioRxiv as Lone et al., 2025), we separated the KMT2A and KMT2F data. We added additional experiments to strengthen the conclusions regarding KMT2F function.

While the KMT2F manuscript was under review in *PLOS Biology*, we continued to investigate the role of KMT2A in rDNA transcription and substantially expanded this work. In the present KMT2A study (Lone and Tyagi, 2025, attached as supplementary file), we demonstrate through ChIP analyses upon KMT2A knockdown that H3K4 trimethylation marks on rDNA were mostly unperturbed. Instead, KMT2A regulates rRNA transcription by recruiting a histone lysine acetyltransferase, and its depletion leads to stalling of RNA polymerase I at the spacer promoter. (However, to fully understand the significance of this finding may require further work.)

Once we received the reviews and noted that the Reviewers specifically requested an assessment of KMT2A function at rDNA, we informed the Editor—in the spirit of full disclosure—that we were preparing a separate manuscript addressing this question and intended to submit it for peer review. The Editor emphasised that the Reviewers' requests to assess KMT2A's role in rDNA regulation were important for demonstrating generality across KMT2 complexes, and advised addressing these points in the current manuscript in *PLOS Biology*.

While we fully appreciate the Reviewers' and Editors' intent to strengthen the manuscript and maximise its impact for the scientific community, we also note that the requested experiments represent a substantial body of work for the current hypothesis. Moreover, based on our prior experience, presenting the findings as two complementary but distinct studies provides greater conceptual clarity, allows deeper mechanistic insight into each KMT2 complex, and avoids the confusion that arose from a combined presentation, as in Lone et al., 2024, while still ensuring that all relevant information is ultimately made available to the scientific community.

We are submitting the second manuscript here for the Reviewers' information. The manuscript was reviewed in the journal iScience but requires further revision.

We are confident that, with the guidance of the Reviewers, the Editor, and the Scientific Editor, we can arrive at the strongest possible presentation of these findings.

REVIEWS:

Reviewer #1:

Specific comments:

1) Figure 1A: In contrast to what the authors state (page 16), KMT2A appears nucleolar but KMT2F appears peri-nucleolar. The distribution of DAPI signal is also very different in the two snapshots. Can this be in any way related to the somewhat distinct genomic distribution profiles of these proteins shown in Figure 1B-E?

We thank the Reviewer for their comment. We have replaced/ deconvoluted the images to reduce background noise, and the image quality has now improved to highlight the nucleolar localization. We have adjusted the DAPI signal in the KMT2F panel, which now appears appropriate.

In addition, we have included an experiment where KMT2F-GFP was expressed (Fig. S1E) in HEK293 cells with B23-dsRed. We observed it to be exclusively nucleolar.

To address potential differences across cell lines (for example, HEK293 cells), we performed immunofluorescence analyses using different nucleolar markers, including UBF, which marks the fibrillar compartment of the nucleolus, Fibrillarin, which marks the dense fibrillar component, and B23, which marks the granular compartment. In all cases (Fig. S1), we consistently observed nucleolar localization of the proteins, thereby ruling out differential genomic distribution along the rDNA locus as an explanation for the observed patterns. We note, however, that

immunofluorescence has inherent technical limitations that make it challenging to resolve the differential distribution of these proteins across individual rDNA repeats.

2) Figures 1 and 2: while the custom assembly genome used in this study has been validated by previous reports, as rDNA is a repeat genomic segment, it is difficult to quantitatively interpret the observed enrichments compared to the rest of the genome. The authors could strengthen their observation that KMT2A/F, PIC and H3K4me2/3 are enriched at the rDNA locus by:

- Analysing their ChIP-seq data on the now available Telomere-to-Telomere (T2T) genome build.

We thank the reviewer for this comment. We have now analyzed the ChIP-seq data using the T2T genome assembly specific for rDNA and observed a similar pattern of binding across the rDNA locus for the different proteins and histone modifications examined (see Reviewer Figure 1).

However, the visualization of rDNA-associated sequencing data on the standard T2T assembly remains challenging, as a typical rDNA chromosome contains approximately 70 highly similar repeats, making it difficult to clearly visualize binding across all repeats simultaneously. As analytical approaches for T2T-based rDNA analysis are still evolving, we are currently refining our pipeline to enable repeat-resolved visualization of factor occupancy across individual rDNA units on all rDNA chromosomes. This approach will facilitate a more precise assessment of the activity status of each rDNA repeat, distinguishing active from inactive units.

- In light of these analyses, more thoroughly illustrate where else in the genome KMT2A and more importantly KMT2F bind, and quantify how specific binding of the KMT2s is to the rDNA locus relative to the rest of the genome (a semi-quantitative attempt to do this is

presented in the qPCR experiments, but this could more generally quantified genome-wide by exploiting the seq data).

This is an excellent suggestion, and we have now performed the quantitative genome-wide analysis of the ChIP-seq data. As shown in Fig S4A-B, consistent with previous observations, RNA Pol I binds exclusively to the rDNA repeats, while a small percentage of UBF goes to genomic loci other than rDNA (Zenter et al., 2011, PMID: 21355038). In addition to observing KMT2A/F on promoter and gene body, we also observed significant levels of these proteins and modifications on the rDNA (Fig. S4C-F).

- Directly comparing the distributions of KMT2A/F with H3K4me2/3, which would show, it appears, that the histone marks correlate with the distribution of KMT2F but not KMT2A at the rDNA locus.

We thank the reviewer for this comment. We have been trying to find a way to perform a correlation analysis comparing the distributions of KMT2A/F with H3K4me2/3 before and now, upon receiving this comment. We discussed this problem with some statisticians as well. However, we have been unable to find a method to do this. Hence, we have removed this statement from the text.

- Providing negative controls. For Figure 1, this could be the distribution analysis of one KMT2 family protein and/or associated factor that is NOT enriched at rDNA. For Figure 2 this could be the distribution of a histone modification that is not enriched at rDNA (H3K4me1 for example? or other lysine methylations such as K27me?)

We thank the Reviewer for this useful suggestion. In order for this analysis to be meaningful, we decided to use the available ChIP-seq data in the same cell line, i.e., HEK293. We used the data for DOT1L and the epigenetic mark deposited by the DOT1L, H3K79me2, as a negative control

for our analysis (Miyamato et al.,2020, PMID: 32997997; Schachner et al., 2021, PMID: 33589837). As shown in Fig. S2, both DOT1L and H3K79me2 show negligible binding to rDNA in contrast to their robust genomic binding described in the reference.

We were unable to find the data for KMT2C ChIP-seq in HEK293.

3) In Figure 3, the the sub-panels "a" and "b" shown in several main panels are not clearly explained. Could the authors comment on these?

We have now explained this in the figure legend.

4) In Figure 3A, the information provided in the cartoon regarding the interaction between KMT2A/F and the PIC is inferred by the authors from the results in Figures 3-5. For clarity, I would advise the authors to show their model figure after the results that support it and not before.

The purpose of including the cartoon in Fig. 3A was to familiarize readers with the various subunits of the RNA Pol I complex. In the context of the present study, the precise positioning of KMT2A/KMT2F within this complex is not yet known. We have therefore removed them from the cartoon and now depict only the RNA Pol I subunits.

5) Figure 4: In the main text, references to SuppFig4G and 4F are inverted.

We have corrected it now.

6) In Figure 4, the authors measure 45S rRNA following RNAi treatment against different KMT2 proteins, and conclude that KMT2B but not KMT2C control rRNA levels. However, the authors do not show evidence that their RNAi experiments are working (i.e that the KMT2B and C indeed display reduced levels as expected). Also, why did the authors exclude KMT2D from their analysis? And how specific do they think is the effect of KMT2A/F on rDNA if 3 out of 4 tested KMT2s have similar effects? In light of this data, it is difficult to understand why the study (as highlighted by the manuscript title) focuses on KMT2F only.

We thank the reviewer for this comment. We have now included data showing the transcript levels of KMT2B and KMT2C following depletion of these proteins (Fig 4A). We have also included WBs for KMT2A/2B/2F and WDR5 upon their knockdown (Fig S6C).

At the outset of our analyses, we followed the widely used three–subgroup classification of the KMT2 family, in which KMT2A/2B, KMT2C/2D, and KMT2F/KMT2G are grouped based on the shared and unique components of their respective multiprotein complexes. (Smith et al., 2011, PMID: 21460034). KMT2A and KMT2B most closely resemble the Drosophila Trithorax protein and form complexes with Menin, a tumour suppressor protein. In contrast, KMT2C and KMT2D assemble into complexes containing Pax transactivation domain–interacting protein (PTIP), the nuclear receptor coactivator NCOA6, and the H3K27 demethylase UTX. Human KMT2F/KMT2G are most similar to the yeast Set1/COMPASS complex, with WDR82 serving as a unique component of the KMT2F/KMT2G complexes that does not associate with KMT2A–D complexes. Based on above broad classification, to investigate the functional contributions of these KMT2 family members, we used siRNAs to deplete KMT2F (from the KMT2F/KMT2G group) and KMT2C (from the KMT2C/KMT2D group).

When KMT2A depletion showed an effect on rRNA, we also targeted KMT2B to validate the above classification further and strengthen our findings. Since KMT2C depletion did not affect rDNA transcription in our assays, we did not prioritise KMT2D for rDNA-focused analyses. Lack of suitable antibodies of KMT2D has also limited more extensive characterization to date.

Although the study is narrated in the present sequence, the transcription assays were performed first. These experiments already indicated that KMT2F regulates rDNA transcription in a SET domain–dependent manner, whereas KMT2A does not (Fig. 4). As explained earlier, we did perform detailed analyses on KMT2A, which revealed that KMT2F and KMT2A regulate rDNA transcription through distinct mechanisms.

7) **In Figure 5, one assumes that the shRNA tool targeting KMT2F used in this figure was not used in previous figures because it was incompatible with rescue constructs. The authors should clarify this point.**

The KMT2F siRNAs provide better knock-down but are expensive to use in ChIP experiments where a large number of cells are required across multiple replicates.

We used two shRNAs to knock down KMT2F. #1 targets the gene, while #2 targets the 3'UTR of KMT2F(Malik et al., 2023, PMID: 37379335). For ChIP experiments, we used both shRNAs to achieve high knockdown efficiency. In experiments related to Fig. 7C, we used shRNA #2 to protect the KMT2F constructs. We have now included this information in the methods.

8) Figure 5 and 6 show a striking effect of KMT2F loss on the epigenomic landscape at the rDNA locus and the formation of the pre-initiation complex. This is the most important finding in this study, and deserves to be strengthened. Furthermore, an additive/synergistic effect of KMT2A and F overexpression on rRNA expression is shown

in Figure 6, it would have been important to evaluate the status of the epigenome and PIC. To do so the authors could:

- Evaluate the epigenomic and PIC status on KMT2A knockdowns as well as control knockdown (KMT2C for example).

We appreciate that the Reviewer intends to strengthen our results and bring out the best results for the scientific community. However, the Reviewer would also be cognizant of the fact that these are a lot of experiments to perform for the presented hypothesis.

As explained above, we have evaluated the epigenomics and PIC status on KMT2A knockdown, shown in the attached manuscript (Lone and Tyagi 2025). KMT2A knockdown does not affect H3K4 trimethylation marks on rDNA but shows a drastic reduction of acetylation levels at H3, including at H3K9. We propose that KMT2A regulates rRNA transcription by recruiting a histone lysine acetyltransferase, and its depletion leads to stalling of RNA polymerase I at the spacer promoter.

As suggested, we have also included KMT2C data, including the epigenomic and PIC status on its knockdown (Fig S8 E-I, S9 C-D). This was not an easy task, as the evaluation of the knockdown of this 500 KDa protein by WB did not work for us and despite extensive shRNA treatment, the chromatin levels of KMT2C reduced modestly on the positive promoter. Hence, we conclude that KMT2C knockdown does not affect either the H3K4me2/me3 status nor the PIC formation on rDNA within our experimental conditions.

- Evaluate the epigenomic and PIC status upon (co-)overexpression of KMT2A/F.

We thank the reviewer for this suggestion. Given that both KMT2A and KMT2F are regulating the rDNA transcription by distinct mechanisms (Fig. 4), in our opinion, to unravel the synergistic effect of both proteins would require a more detailed study, including the use of a TA domain mutant of KMT2A and a SET domain mutant of KMT2F, and their combinations. While this is an important question to answer, we feel this is best undertaken as a future separate study.

- Evaluate the impact on the epigenomic and PIC status upon rescue with mutated forms of KMT2F (if possible, considering the construct sequences).

This experiment was included in the manuscript and is referenced in the comment below. We had previously shown the PIC status for KMT2F and KMT2F mutants in Supplementary Fig. 7; this has now been moved to the revised Fig. 7. In this experiment, we demonstrate that expression of exogenous wt KMT2F in the background of knockdown of endogenous KMT2F maintains RNA Pol I and UBF levels at the rDNA promoter. In contrast, expression of a catalytically dead KMT2F mutant fails to restore these levels. Consistent with our working hypothesis, these data provide compelling evidence that the KMT activity of KMT2F—and by extension the H3K4me3 mark—is required for proper PIC formation.

9) Two paragraphs of the main text refer to a supplementary figure (7). These results are convincing and interesting, although negative. For clarity, I would advice the authors either include this information as a main Figure or reduce the respective text.

As described above, part of these findings have moved to the main Fig 7, while the interaction with the KMT2F RRM domain remains in the supplementary figure (Fig. S9 E).

Reviewer #2: Comments for PBIOLOGY-D-25-00691

Major points

1) In the introduction, the authors state that KMT2A and KMT2F deposit H3K4me2/3 at gene promoters, and that loss of these enzymes and modifications "leads to a widespread decrease in transcriptional output" by RNA polymerase II (RNAP2). Then, the simplest explanation of their data is that loss of KMT2A/F leads to a decrease in the expression of factors that regulate rRNA transcription and/or processing, meaning the decrease in rRNA transcription is not by direct epigenetic regulation at rDNA. Can the authors show that there isn't broad genic transcriptional re-programming that can explain the decrease in rRNA transcription in cells lacking KMT2A/F?

We thank the Reviewer for raising this important question. We have carefully considered this issue, and the interaction studies examining KMT associations with the RNA Pol I machinery were undertaken both to establish their physical proximity to Pol I and to exclude indirect effects arising from global transcriptional reprogramming.

To directly assess whether broad transcriptional changes could account for the observed effects on rDNA transcription, we analysed our recently published RNA-seq datasets (Malik et al., 2023, PMID: 37379335) following depletion of KMT2A or KMT2F, alongside the corresponding control

conditions. Pathway-level analyses focusing on ribosome biogenesis revealed no significant alterations upon depletion of either protein (Fig. S5G-H). Together, these findings support the conclusion that the effects of KMT2A and KMT2F on rDNA transcription are likely to be direct rather than secondary consequences of widespread transcriptional reprogramming.

2) rRNA transcription is strongly linked to cell proliferation. The authors need to show whether KMT2A/F loss affects cell viability/growth.

Because we depleted these proteins for a relatively short time window (72 h), we did not observe any drastic effects on cell proliferation or cell cycle progression. Consistent with this, our previous study (Malik et al., 2023, PMID: 37379335) demonstrated near-normal cell cycle progression in inducible KMT2A knockout cells. Similarly, it has been reported that cells remain viable following short-term depletion of KMT2F (Wang et al., 2017, PMID: 29138278). As our experiments were also conducted within a limited depletion window, the lack of detectable effects on cell proliferation is consistent with these earlier observations.

3) For Figures 3, 4 and 5 and several supplemental figures, Western blots validating shRNA efficacy and transgene re-expression are essential to interpreting this data. Also, it is important to also show H3K4me2/3 levels in these different cell lines.

We have included the western blots in several figures (Fig S1, S6)

In our effort to keep the transgene expression as close to the endogenous expression, we had selected cell-clones accordingly (Ali et al., 2014, PMID: 24880690). However, this decision makes it very difficult for us to detect expression over the wild-type protein by WB. We used IFS to demonstrate that the cells are expressing the said proteins as also shown previously (Chinchole et al., 2022, PMID: 36111497; Chodisetty et al., 2024 PMID: 39661677). Here, we used paraformaldehyde fixation to visualize the nuclear localization of exogenous proteins by anti-

FLAG antibody. The same is shown in Fig S7A. We had previously demonstrated the expression of these transcripts in knockdown cells in Fig. S6G-I.

The H3K4me2/3 blots are shown in Fig. S8D.

4) For co-immunoprecipitations and pulldowns, it would strengthen the authors arguments/model to show positive controls such as RNAP2 to show how strong the interactions are relative to known binders.

This is an excellent suggestion, and we have included the data for the same in Fig. S6B.

5) While the use of different cell lines is typically helpful to interpret the significance of the data, here many datasets are compared across cell lines, but all cell lines have different epigenetic backgrounds, proliferation rates, and immortalization methods, each of which can affect rDNA transcription. For example, their blots in Figure 3 were done largely using HeLa lysates to demonstrate KMT interactions with RNA Pol I machinery, but genomic and PCR studies were done largely in HEK293 or U2OS cells. Additionally, in Figure 1 and Figure S1, KMT2A and KMT2F localizations appeared cell line-dependent, but the only validation for their localization was done in U2OS cells (Fig. S1b). To increase confidence in these phenotypes, it will be important to repeat some of these experiments consistently in the same cell lines.

We intended to demonstrate a cell-line independent role for KMT2A and KMT2F in rDNA transcription. However, we appreciate the Reviewer's suggestion. As the majority of our genomic analyses were performed in HEK293 cells, we have strengthened our conclusions in these cells

by incorporating additional experiments like, IFS and its validation (Fig. S1 C-D), co-expression of KMT-GFP with B23-dsRed (Fig. S1 G) and transcript analyses (Fig. S6D-E) experiments. Notably, the results obtained here are consistent with our previous results.

6) For many figures, inappropriate statistical tests were used.

We have now changed the statistical tests as shown in the figure legends.

Minor points

1) The nucleolar localization of KMT2s in supplemental figure 1 is not convincing. This is also concerning because this looks very different than what is shown in main Figure 1.

We thank the Reviewer for the suggestion. As the Reviewer rightly pointed out, different cell lines have different states of rDNA transcription, and that would affect their nucleolar sizes. Hence, we have now either deconvoluted or shown magnified images to highlight their nucleolar localisation. Additionally, the KMT2-GFP staining in HEK293 (Fig. S1G), added as part of the Reviewer's comment above, shows very clear nucleolar staining.

2) The authors state "We found that WDR82 showed higher enrichment in the transcribed unit (Supplementary Figure S2), consistent with KMT2F binding." But that is a vast overstatement based on the data. The presence of WDR82 at rDNA does not mirror KMT2F strongly at all. And you certainly can't state that KMT2F binds WDR82 from this data.

We have modified the text as follows:

We found that WDR82 showed higher enrichment in the transcribed unit (Supplementary Figure S2), indicating a possible role at the rDNA locus.

3) The ChIP-PCR and ChIP-seq results don't entirely align, and it is not clear at all how or what statistical tests are use for all ChIP-PCR experiments.

We performed a quantitative analysis of the ChIP-seq signal corresponding to each primer used for qPCR and compared these values with the ChIP–qPCR data (Fig. S4A–D). Overall, the two datasets are broadly concordant, although some discrepancies are observed. For example, primer #6 shows variation between the ChIP–qPCR and ChIP-seq datasets for KMT2F, whereas the remaining regions display good overall correlation.

We would also like to note that analysis of protein occupancy at the rDNA locus is technically challenging and associated with inherent limitations due to its highly repetitive nature. In the present study, ChIP-seq signals were aggregated across ~400 rDNA repeats and presented as collated data. An ideal future approach would involve visualization of factor occupancy across individual rDNA units on all rDNA-bearing chromosomes.

Also we have now changed the statistical tests as mentioned in the legends.

4) For H3K4me2/3 in Fig. 2, it is hard to tell whether there is an enrichment at rDNA or whether the mark is simply present at rDNA. Meaning, is this mark enriched relative to other genomic loci? Also, while these marks look similar from ChIP-seq but not by ChIP-PCR. How can you reconcile this?

To address the Reviewer's query, we have now performed several additional experiments. (i) We carried out genome-wide analyses of these histone marks together with their corresponding proteins and observed significant enrichment at the rDNA locus (Fig. S5C–F). (ii) We included H3K79me2 as a negative control histone mark and show that, in contrast to H3K4me2/me3, H3K79me2 is not enriched at rDNA (Fig. S2). (iii) To assess concordance between the ChIP-seq and ChIP–qPCR datasets, we quantitatively analysed the ChIP-seq signal over the qPCR target regions and compared these values with the ChIP–qPCR results (Fig. S4); the two approaches show good agreement.

5) The authors state "Again, we validated our findings by ChIP analyses and observed consistently high enrichment of these marks on RNA Pol I promoter and IGS region in HEK293(Figure 2B-C)." However, this should read "rDNA promoter"

We have corrected this mistake.

6) For Fig. 3C, It is hard to interpret the data because the KMT2A-SET positive control and input are on a separate blot, making it difficult to interpret how strongly WDR5 binds to RPAs.

The experiment was designed to include all RPAs along with the positive control, and all pull-down assays were performed side by side across replicates. SDS–PAGE, transfer, and blot development were likewise carried out in parallel. Nevertheless, we limit our conclusion to stating that *WDR5 specifically interacts with RPA49 and RPA34*. The apparent strength of these interactions is influenced by the levels of GST-tagged protein expression (RPA versus KMT2A-SET), despite our best efforts, they are not equal. Consequently, we refrain from drawing any comparative conclusions regarding interaction strength.

7) For Fig. 3E, is the interaction with RPOL1 components dependent on solely KMT2A or KMT2F? Can you do co-IPs in KMT2A or KMT2F siRNA?

Based on our following studies (i) KMT2F-RRM interaction study (Fig. S9E), where a small domain of KMT2F is sufficient to pull down the RNA Pol I complex, (ii) KMT2F wt Vs mutant pull downs (Fig 7A, C), where the interaction of KMT2F is retained in both cases and (iii) our overexpression studies (Fig. 7D), where expression of either protein can stimulate rRNA transcription, we provide sufficient indirect evidence that KMT2A or KMT2F can independently interact with RNA Pol I components.

Importantly, we have not claimed that this interaction is exclusively dependent on a single KMT2 family member. In light of these findings, and considering the experimental effort required, we believe that the suggested experiment would not add significant support to our central hypothesis and therefore respectfully chose not to pursue it.

8) For Fig. 4A, the authors state "The transcript levels of KMT2A, KMT2F and WDR5 decreased by more than 60%..." but they need to show protein levels.

We have now included the blots in Fig. S6C.

9) The authors state "Remarkably, consistent with the role of SET domain of KMT2F in our rRNA transcript analyses, knockdown of KMT2F resulted in drastic reduction of H3K4me2 and H3K4me3 levels on the whole rDNA loci (Figure 5C-D, S6B-C)," but how are the global levels of H3K4me2/3 affected? Have you performed western blots or ChIP-seq or PCR at other loci?

Besides showing ChIP-qPCR for positive and negative promoters (Fig. S8A-C, S9A-B), we have now included the western blots showing the global levels of these histone modifications upon KMT2F knockdown (Fig S8D).

As we aimed to maintain consistency by using the same cell line (HEK293) for rDNA genomic analyses, we searched for publicly available ChIP-seq datasets examining H3K4me2 and H3K4me3 under KMT2F-depleted conditions in HEK293 cells. However, no such datasets are currently available, which limits our ability to extend the analysis for these histone modifications in this cell type. We appreciate the reviewer's suggestion and plan to strengthen this aspect in future work by performing analogous studies in mouse embryonic stem cells.

10) The results from Fig. 5C are difficult to interpret because the ChIP seq and ChIP PCR are not concordant. Also, the three biological replicates shown are very inconsistent. To definitively show this, you'd need to also look at K4me2/3 levels in KMT2A-depleted cells.

Given that these are biological replicates, we feel that the results are consistent with each other (except primer #1, where one reading is higher than the other two). We have also included the quantitative analysis of the ChIP-seq signal over the qPCR amplicons, and H3K4me2 shows the best correlation (Fig. S4C).

We have looked at the H3K4me2/me3 levels in KMT2A-depleted cells (Fig. 2, Lone and Tyagi 2025), and we have observed a decrease in H3K4me2 on Primer #1-3 while H3K4me3 decreases only in Primer #1. These observations are very distinct from those made in KMT2F-depleted cells.

11) The authors state "….indicating that by depositing H3K4me3 mark, KMT2F prevents the heterochromatinization of rDNA and keeps it open for transcription." This is an over interpretation of the data given what is shown.

We have now changed the statement to:

KMT2F prevents the repression of rDNA and keeps it open for transcription.

12) Based on the results of Fig. S7C, the authors state "our results imply that the KMT2F may be recruited to the rDNA promoter, indirectly through WDR82." But they should either test this using knockdown of WDR82 or express an RRM-deletion mutant of KMT2F to test this.

This statement indicates a possibility, based on the results obtained. However, based on the suggestion, we have modified it further to increase the suggestive element.

Given that RRN3 binds active RNA Pol I specifically at the rDNA promoter, our results support a model in which KMT2F is recruited to the rDNA promoter, most plausibly through an indirect interaction mediated by WDR82.

Reviewer Figure 1: Chromatin immunoprecipitation–sequencing (ChIP-seq) profiles depicting the binding of RNA polymerase I (RPA194), UBF, WDR82, WDR5, KMT2A, KMT2F, H3K4me2, and H3K4me3 across the entire human rDNA locus are shown, together with DOT1L and H3K79me2. Read alignment was performed using the T2T genome assembly modified for rDNA (hs1-rDNA). ChIP enrichment for each factor is presented as the ratio of ChIP-seq signal to the corresponding input DNA signal, with the vertical axis in each track representing enrichment relative to input. In the hs1-rDNA annotation, George *et al.* (2023) excised 9.18 kb from the end of the rDNA repeat and appended it to the beginning of the repeat; consequently, the 47S promoter is positioned 9.18 kb from the start of the repeat. Accordingly, the x-axis is displayed from −9.18 to 34.8 kb, with the 47S promoter designated as position 0.

T2T

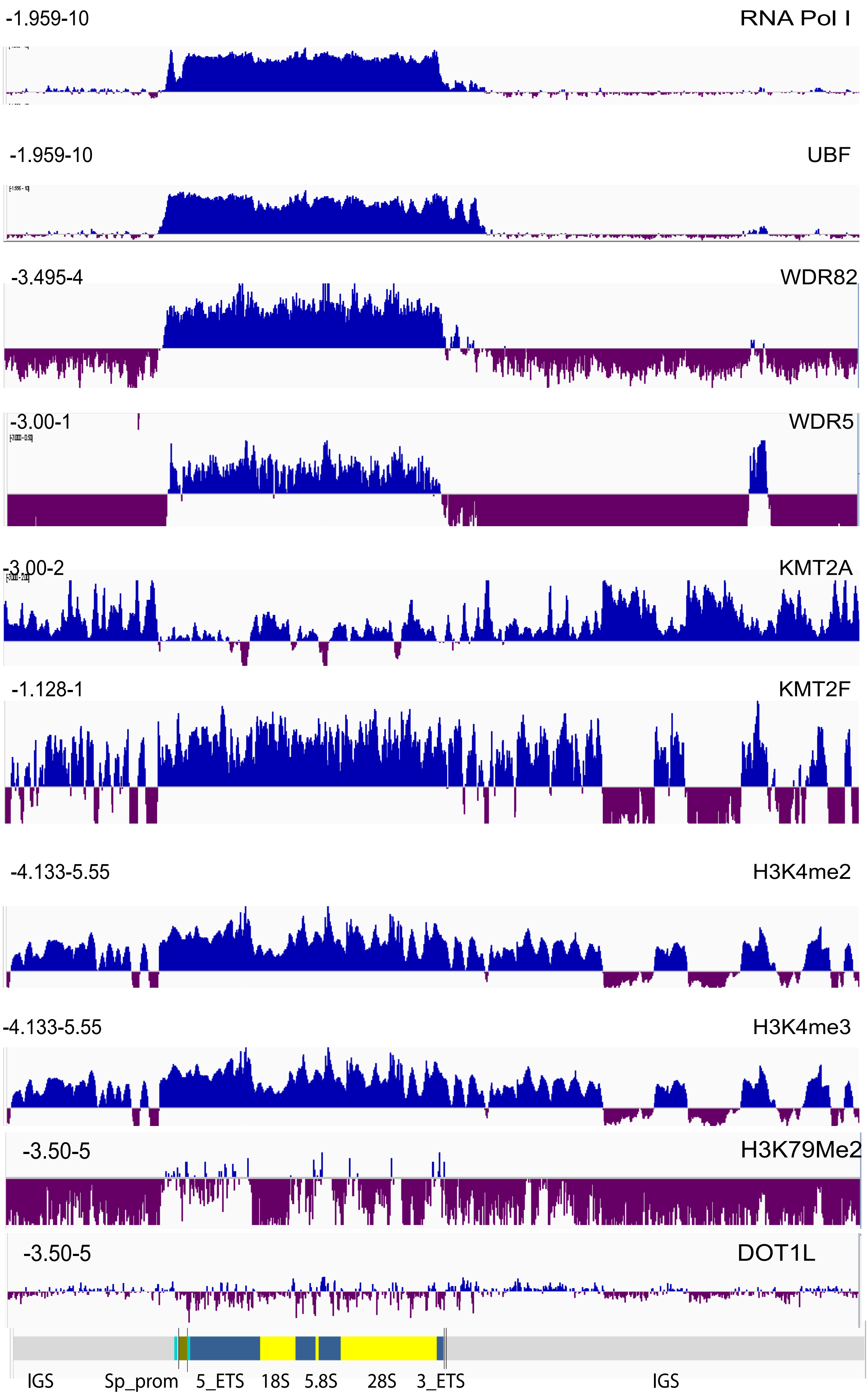

Lone Reviewer Figure 1
