# Supplementary material for: The KMT2F histone methyltransferase interacts with the RNA polymerase I machinery to promote ribosomal RNA transcription
Source: PLoS Biol. 2026 May 7;24(5):e3003785. doi: 10.1371/journal.pbio.3003785 (PMC13178980; doi:10.1371/journal.pbio.3003785)
Supplement: Attachment — Submitted filename: Response to Reviewers.docx [file pbio.3003785.s016.docx]

**Response to Reviewers**	Reviewer: they/them

We thank all the reviewers for their careful evaluation of our manuscript, constructive suggestions, and valuable feedback. These revisions have strengthened the quality and clarity of our findings, improved the overall presentation, and made the study more accessible and convincing to the readers.

**Reviewer #1: The authors have made substantial efforts to clarify their initial version of this manuscript and to provide compelling additional data and analyses.
Given these additions, I agree with the authors that it would be unreasonable to go even further within the scope of this study.
I thus recommend this manuscript in its current form for publication in PLoS Biology and congratulate the authors for their excellent work.**

We are grateful to Reviewer 1 for their thorough and thoughtful evaluation of our manuscript and for providing valuable and constructive feedback. These revisions have helped improve the clarity and overall quality of our work.

**Reviewer #2: In this manuscript, the authors sought to investigate the role of H3K4me3 at ribosomal DNA (rDNA) during transcription. They identify 1) H3K4me3 appears to be present and potentially enriched at rDNA, 2) KMT2F as the primary methyltransferase of H3K4me3 at rDNA, and 3) loss of KMT2F and H3K4me3 decreases rDNA transcription.

I feel the authors have sufficiently addressed our major concerns, albeit while raising several other smaller issues:

1) In supplemental figure 1, co-localization of NPM1 and KMT2A/F are more convincing than their previous data and consistent with their statement that these factors localize to the nucleolus. While the images appear to show a more substantial decrease in KMT2 levels than their western blots, there is less staining in the siKMT conditions. Intriguingly, given their proposal of the role of KMT2 in the regulation of rDNA transcription, the organization of the nucleolus appears unchanged upon KMT2 knockdown.**

We thank the Reviewer for their comments. The observed decrease in KMT levels in both Western blot and immunofluorescence (IF) can be interpreted in the context of the methodologies used. Western blot analysis here measures protein levels in whole-cell lysates, thereby reflects the total cellular pool of KMTs. In contrast, IF is used here to specifically examined the protein localization of KMTs within the nucleolus, and each cell can be visualized independently. As a result, even under KMT2-depleted conditions, residual protein levels from other cellular compartments may still be detectable in Western blot, whereas IF more clearly reveals localized changes within the nucleolus.

We have understood the Reviewer’s concern here. As the Reviewer is aware, the nucleolus is made up of Fibrillar compartment, Dense Fibrillar compartment, and Granular compartment, and we are currently in the process of analysing how these compartments change with respect to each other as well as in their overall organization of the nucleolus upon KD of our proteins, by employing 4-colour high-resolution microscopy. Therefore, at present, we do not wish to make a statement about the change in the organization of the nucleolus till we have completed our detailed analyses. We hope to report these findings in the near future.

**2) Another issue we raised was the fact that loss of KMT2 may change transcription of ribosome biogenesis factors. To address this, the authors re-analyzed previously published RNAseq data from HeLa cells with knockdown of KMT2A/F. Of note, HeLa cells were not the model used for many of the other experiments. While it is very surprising to see that the nuclear enrichment scores for myriad ribosome biogenesis GO terms appear to be exactly the same at 0, it does appear to suggest that the majority of pathways regulated by KMT2 are independent of ribosome biogenesis. Of note, it is unclear what the "Significance" color codes are for in Supp. Fig. 5G,H. Also, I would request the authors delineate what statistical cutoffs were to generate these plots.**

First the clarification: we performed the RNA-seq in the U2OS cells in our previous study and reanalysed it here to address the Reviewer’s question. Notably, we have performed most of our RNA and IFS experiments in U2OS cells.

We thank the reviewer for highlighting the need for clarification regarding the statistical cutoffs and colour coding. As described in the Methods section, we applied the Benjamini–Hochberg correction to determine statistical significance, and genes with an adjusted *p*-value < 0.05 and a fold change ≥ 1.0 were considered differentially expressed. Of note, none of the ribosome biogenesis GO terms appeared significant and had to be manually curated.

Regarding the colour scheme, a legend has been provided alongside the figure: red denotes top upregulated terms, blue indicates top downregulated GO terms, and orange represents no significant change, hence marked zero. We acknowledge that the additional black and silver colour coding may have caused confusion, and we have removed these elements to improve clarity

**3) To determine whether KMT2A/F and H3K4me3 are present or enriched at rDNA, the authors re-analyzed ChIPseq data to identify the proportion of these factors or modifications across the genome (Supp Fig 5A-F). While their data showed that KMT2A/F and H3K4me3 are present at rDNA, among other loci, it is slightly difficult to interpret because these bins (intergenic, gene bodies, rDNA, promoter) are not equivalent in the proportion of the genome. For example, gene bodies encompass ~1-2% of the genome, yet ~25% of total H3K4me3 and KMT2 are present in these regions. Thus, it is unclear how to interpret the data, which would be more convincing and interpretable if scaled accordingly.**

We thank the reviewer for their comment. We had considered the genomic size of these bins as we had normalized the read counts from these regions with respect to their genomic size. We have added a line in our methods section to clarify this further.

Please note that KMT2/H3K4me3 has been reported to be highly enriched on the promoters (which also encompass a very small portion of the genome 1-2% or less ) in previous studies (PMID: [40196527](https://pubmed.ncbi.nlm.nih.gov/40196527/), PMID: [28157506](https://pubmed.ncbi.nlm.nih.gov/28157506/)), and our analyses reflect these findings. Unfortunately, due to annotation issues in the rDNA assemblies, we were unable to make a distinction between the first exon and the remaining gene body, as this may likely change the distribution further. Moreover, as rDNA was not annotated before, readings may have been adjusted accordingly, but we feel that overall they reflect the KMT2 distribution as reported before.

Finally, we sincerely thank the Reviewer for their careful evaluation of our manuscript and for providing insightful suggestions to improve the robustness of our study.
